# Clinical effectiveness of pit and fissure sealants in primary and permanent teeth of children and adolescents: an umbrella review

**DOI:** 10.1007/s40368-024-00876-9

**Published:** 2024-03-15

**Authors:** S. Amend, C. Boutsiouki, J. Winter, D. Kloukos, R. Frankenberger, N. Krämer

**Affiliations:** 1 Department of Paediatric Dentistry, Medical Centre for Dentistry, University Medical Centre Giessen and Marburg (Campus Giessen), Justus-Liebig-University Giessen, Schlangenzahl 14, 35392 Giessen, Germany; 2 Department of Operative Dentistry, Endodontics and Paediatric Dentistry, Medical Centre for Dentistry, University Medical Centre Giessen and Marburg (Campus Marburg), Philipps-University Marburg, Georg-Voigt-Str. 3, 35039 Marburg, Germany; 3 Department of Orthodontics and Dentofacial Orthopedics, University of Bern, Freiburgstr. 7, 3008 Bern, Switzerland

**Keywords:** Clinical effectiveness, Pit and fissure sealants, Primary teeth, Permanent teeth, Children, Adolescents, Umbrella review

## Abstract

**Purpose:**

This umbrella review aimed to critically appraise the evidence published in systematic reviews (SRs) on the clinical effectiveness of sealants compared with each other/the non-use in primary/permanent teeth of children and adolescents with at least 12-month follow-up.

**Methods:**

A systematic literature search on 4 electronic databases was conducted up to January 18th, 2023. Following handsearching, two review authors independently screened retrieved articles, extracted data, and assessed the risk of bias (RoB) using the risk of bias in systematic reviews (ROBIS) tool. Based on a citation matrix, the overlap was interpreted by the corrected covered area (CCA).

**Results:**

Of 239 retrieved records, 7 SRs met the eligibility criteria with a moderate overlap among them (*CCA* = 7.4%). For primary molars, in 1120 1.5- to 8-year-old children, data on the clinical effectiveness of sealants were inconclusive. For permanent molars, 3 SRs found a significant caries risk reduction for sealants versus non-use (≤ 36-month follow-up). There was insufficient evidence to proof superiority of sealants over fluoride varnish for caries prevention (3 SRs), and to rank sealant materials according to the best clinical effectiveness in permanent molars. One study was rated at low and 6 at high RoB, which did not allow for a valid quantitative synthesis.

**Conclusion:**

Considering the limitations of this umbrella review, sealants are more effective for caries prevention in children’s permanent molars compared to no treatment. Future well-implemented RCTs are needed to draw reliable conclusions on the clinical effectiveness of sealants in primary and permanent teeth of children and adolescents.

**Supplementary Information:**

The online version contains supplementary material available at 10.1007/s40368-024-00876-9.

## Introduction

Dental caries in primary and permanent teeth is one of the most prevalent diseases worldwide and may affect all tooth surfaces (Collaborators et al. 2020). Although 12.5% of all tooth surfaces are occlusal, the morphological complexity of these surfaces contributes to the development of more than two-thirds of the total caries experience of children (Ripa 1973). Susceptibility to plaque accumulation and food retention is the reason for the increased occlusal caries incidence (Bagherian and Shirazi 2018). In particular, the occlusal surfaces of first permanent molars and, to a lesser degree, those of second permanent molars are known to be at an increased caries susceptibility in the first years after eruption (Carvalho 2014).

Dental sealants were introduced in the 1960s as resin-based materials to help prevent dental caries, mainly in the pits and fissures of occlusal tooth surfaces, acting as a physical barrier to prevent caries initiation and progression in pits and fissures (Ahovuo-Saloranta et al. 2017). It involved the application of a thin layer of material on the occlusal surface after acid pre-treatment (Welbury et al. 2004). Later on in the 1970s, glass ionomer-based sealants were suggested as an alternative due to its advantage of fluoride release and to its chemical adhesion without acid pre-treatment (Mejàre et al. 2003).

Fissure sealants can be classified into resin-based sealants, glass ionomer-based sealants and hybrid sealants (Ramamurthy et al. 2022). First, methyl methacrylate or cyanoacrylate cements were used until resin-based sealants with bisphenol A-glycidyl methacrylate (Bis-GMA) were invented (Bowen 1982). Based on the content and the polymerisation method, four generations of resin-based sealants can be defined: UV light polymerised, autopolymerised, blue visible light polymerised and fluoride-releasing (Ahovuo-Saloranta et al. 2017). The first generation showed degradation in the oral cavity and is no longer available (Ahovuo-Saloranta et al. 2017). Glass ionomer-based sealants are widely used due to their fluoride-releasing property (Welbury et al. 2004). In addition, these sealers are less sensitive to moisture but have poorer retention rates on teeth compared to resin-based sealants (Simonsen 2002). Glass ionomer-based sealants can be conventionally (chemically) cured, or resin modified. The resin-modified ones are a combination of glass ionomer cements (GICs) with resin components, which are light-cured (Arrondo et al. 2009). In addition, there are hybrid sealants such as compomers and giomers, whose data on the caries-preventive effect are limited so far (Ahovuo-Saloranta et al. 2017). Compomers are polyacid-modified composite resins and giomers are fluoride-releasing materials made from urethane resins which contain surface-pretreated glass ionomer filler particles (Ramamurthy et al. 2022).

According to the guidelines for the use of pit and fissure sealants published by the European Academy of Paediatric Dentistry (EAPD) in 2004, “*a fissure sealant is a material that is placed in the pits and fissures of teeth to prevent or arrest the development of dental caries*” (Welbury et al. 2004). The caries-preventive effect of fissure sealing may be related to caries incidence level of the population (Ahovuo-Saloranta et al. 2017), type of sealant material (Mejàre et al. 2003), single or repeated sealant applications, follow-up time, type of tooth and jaw, the operator, the content of fluoride in the drinking water (Llodra et al. 1993), and isolation from saliva (Eskandarian et al. 2015). Regarding adverse effects of dental sealants, some concerns have been raised for allergic reactions and estrogen-like effects of resin-based materials including bisphenol A (BPA) (Fleisch et al. 2010; Furche et al. 2013; Kloukos et al. 2013). However, current consensus is that sealants are safe (Ahovuo-Saloranta et al. 2017).

The aim of this umbrella review was to critically appraise the available evidence published in systematic reviews on the clinical effectiveness of pit and fissure sealants compared either to each other or with the non-use of sealants in primary and permanent teeth of children and adolescents over a follow-up of at least 12 months.

## Methods

### Umbrella review protocol and reporting format

The a priori prepared protocol for this umbrella review was registered in PROSPERO, the international prospective register of systematic reviews hosted by the University of York, Centre for Reviews and Dissemination, York, UK (CRD42023391620). During the whole review process, the methods described in the Cochrane Handbook for Systematic Reviews of Interventions (Higgins et al. 2019) and the Preferred Reporting Items for Systematic Reviews and Meta-Analyses (PRISMA) were adopted (Page et al. 2021).

### Umbrella review focused question and PICO(S)

The following focused question was constructed for this umbrella review:

What is the best available evidence of systematic reviews on the clinical effectiveness of different pit and fissure sealants contrasted either to each other or to the non-use of sealants in primary and permanent teeth of children and adolescents over a follow-up of at least 12 months?

Based in this review question, the PICO(S) schema for the included systematic reviews was defined as follows:

*Participants/population (P)*: Pit and fissure sealants placed on occlusal surfaces of primary molars or permanent premolars/molars, which were caries-free (either stated verbatim or referred to as ICDAS-II 0 (Ismail et al. 2007; Pitts 2004)) or affected by initial carious lesions (either stated verbatim or referred to as ICDAS-II 1–3 (Ismail et al. 2007; Pitts 2004)), in children and adolescents up to the age of 19 years.

*Interventions (I)*: Sealants: Any of the following sealing materials was considered as pit and fissure sealant: composite resins, polyacid-modified composite resins (compomers), glass-ionomer cements. Pre-treatment: Different pre-treatments before sealant application were accepted. There were neither restrictions on the personnel conducting the pit and fissure sealing nor on the setting, in which the treatment was performed.

*Comparators (C):* Any other of the pit and fissure sealants mentioned above or no sealant.

*Outcomes (O):* The primary outcomes of this umbrella review were (1) incidence of carious lesions extending into dentine (either stated verbatim or referred to as ICDAS-II scores 4–6 (Ismail et al. 2007; Pitts 2004), Ekstrand, scores ≥ 2 (Ekstrand et al. 1998)) on previously sealed sound primary or permanent teeth (clinical assessment applying visual or visual-tactile criteria); (2) progression of existing initial carious lesions extending into dentine on sealed primary or permanent teeth (clinical assessment applying visual or visual-tactile criteria); (3) success rate, retention rate, (annual) failure rate, survival, longevity of sealants in primary and permanent teeth.

The secondary outcomes were (1) adverse events; (2) influence of pretreatment procedures or type of isolation; (3) clinical treatment time; (4) patient acceptability; (5) bisphenol A (BPA) release; (6) cost/benefit analysis. Secondary outcomes were only considered when they were mentioned in the included systematic reviews.

*Study design (S):* systematic reviews with/without meta-analyses.

### Inclusion and exclusion criteria

To be included in this umbrella review, systematic reviews with/without meta-analyses needed to include primary studies comparing the clinical effectiveness of occlusal pit and fissure sealings with different sealant materials or sealant non-use in primary and/or permanent teeth of children and adolescents over a period of at least 12 months. In this context, the term “*primary studies*” refers to the initial studies included in the systematic reviews meeting the inclusion criteria of this umbrella review.

Any other study types except for systematic reviews with/without meta-analyses were excluded. Further exclusion criteria were systematic reviews with a follow-up less than 12 months, participants aged ≥ 19 years, sealants placed on cavitated dentine carious lesions, sealants combined with restorations in the same tooth, and sealants combined with further caries-preventive measures in single groups.

### Search strategy

An experienced review author (DK) developed a comprehensive search strategy and adequately adapted it for each electronic database, considering the characteristics of syntax rules and controlled vocabulary. The following four electronic databases were searched on 18 January 2023: MEDLINE (PubMed), Embase (Ovid), Cochrane Library, and LILACS. The search was neither restricted to publication date nor to language of the systematic reviews. The reference lists of the included systematic reviews were screened for further eligible studies, which had not been retrieved through online searches, by one review author (JW).

### Selection of the systematic reviews

First, all titles and abstracts of studies retrieved were screened independently and in duplicate by two review authors (CB, SA) using the online platform Rayyan to identify those potentially meeting the inclusion criteria (Ouzzani et al. 2016). If abstracts were not available or information were missing in the abstract, studies were considered for full-text reading as long as the available information seemed to meet the inclusion criteria. Full texts of all studies which were not excluded during title and abstract screening were screened independently two review authors (CB, SA) for eligibility. Systematic review authors were contacted by email as an attempt to gather missing information that could not be located in the report. If full texts could not be retrieved, the respective study had to be excluded. All systematic reviews that had been excluded at full-text stage were recorded along with the reason for exclusion. Disagreements occurring at any stage during the study selection process were resolved by discussion and, if necessary, consultation of a third review author.

### Data extraction

Data extraction was conducted independently by two review authors (CB, SA) and relevant data were added to an Excel spreadsheet (Microsoft Corporation, Redmond, WA, USA) prepared for data extraction, which had been pilot-tested beforehand by the same two review authors by selecting five of the included systematic reviews. The following data were extracted of the included systematic reviews:

- General information: review authors, title, publication year, country, review design, databases screened, risk of bias tool, quality of evidence tool, inclusion and exclusion criteria, follow-up, results of the quality assessment, conflicts, notes.
- Participants: number of participants, age, type of teeth, number of teeth assessed initially/at the final follow-up, caries prevalence, extent of caries.
- Intervention/control: type of pretreatment, type of isolation, type of intervention/control, sealant materials used.
- Outcome measures: caries incidence, retention, adverse events, assessment criteria, reasons for failure, results of meta-analyses.

The extracted data were double-checked by a third review author (JW).

### Calculation of the degree of overlap

The degree of overlap of primary studies being included in several systematic reviews was assessed by calculating the corrected covered area (*CCA*), a validated measure introduced by Pieper et al. in 2014 (Pieper et al. 2014a, b). In brief, a citation matrix was generated and the degree of overlap was computed using the formula $CCA = \frac{N - r}{{rc - r}}$ with *N* indicating the number of included primary studies (double counting permitted), *r* representing the number of index publications, and *c* depicting the number of included systematic reviews. The *CCA* [%] was interpreted as slight (0–5%), moderate (6–10%), high (11–15%), and very high (> 15%) overlap.

### Quality assessment of the included systematic reviews and meta-analyses

Two review authors (CB, SA) assessed the risk of bias in systematic reviews (ROBIS) independently using the ROBIS tool, which consists of three phases:

- Phase 1. Optional assessment of the systematic review’s relevance
- Phase 2. Identification of concerns with the review process including the four domains study eligibility criteria, identification and selection of studies, data collection and study appraisal, as well as synthesis and findings.
- Phase 3. Judgement on the risk of bias in the systematic review (Whiting et al. 2016).

Again, disagreements were resolved by discussion and by consultation of a third party.

### Common effect size estimation

It was foreseen to convert all effect sizes into corresponding Odds Ratios (ORs) with the computer software ReviewManager (RevMan 5; The Cochrane Collaboration, London, UK). Meta-analyses were planned to be conducted in case of limited clinical, methodological, and statistical heterogeneity by including publications at low risk of bias.

## Results

### Results of the systematic literature search

**Fig. 1:**
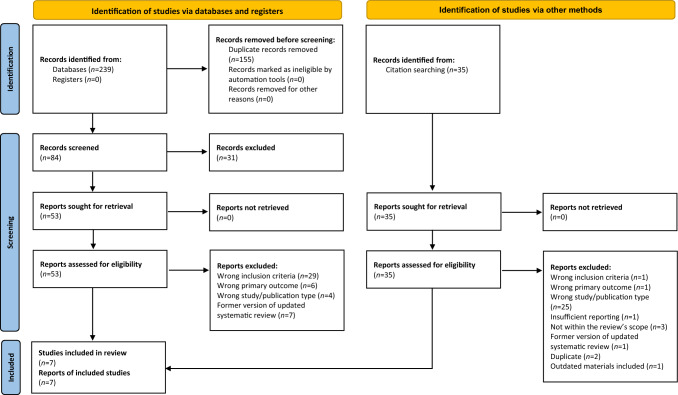
PRISMA flow diagram modified according to Page et al. (2021)

Two hundred and thirty-nine records were identified by the initial systematic literature search on four electronic databases. Appendix 1 shows the search strategy applied for electronic database screening. After duplicate removal (*n* = 155), 84 records were considered, of which 31 had to be excluded after title and abstract screening. Additional 35 records were retrieved by citation searching. In four studies, information about the participants age was not obtained, which is why the included primary studies were retrieved to check the participants’ age (Bagheri et al. 2022; Bagherian and Shirazi 2016, 2018; Beiruti et al. 2006a, b). A sum of 88 records was assessed for eligibility, of which 81 records did not meet the inclusion criteria and had to be excluded at full text reading stage. A consensus-based decision was made to exclude one systematic review due to a lack of reporting on a quality assessment of included primary studies (Condo et al. 2013), and another one because of evaluating partly outdated sealant materials (Llodra et al. 1993). The reasons for exclusion are summarized in Appendix 2. Seven systematic reviews with (*n* = 6) or without meta-analyses (*n* = 1) were finally included in this umbrella review. The process of identifying studies is presented in the PRISMA 2020 flow diagram (Page et al. 2021) (Fig. 1).

### Overlap of primary studies included in the systematic reviews

In the 7 systematic reviews, a sum of 101 primary studies was included if double counting was permitted (Appendix 3). The corrected covered area (*CCA*) amounted to 0.074 (7.4%) with *N* = 101 for the number of primary studies including double counting, *r* = 70 for the number of index publications (rows), and *c* = 7 for the number of index reviews (columns). Therefore, overlapping was moderate for the present umbrella review (Pieper et al. 2014a, b).

### Characteristics of included systematic reviews

The characteristics and results of included systematic reviews are presented in Tables 1, 2, 3. Databases screened for primary studies meeting the inclusion criteria of the included systematic reviews were Biomed Central (*n* = 1), CNKI (*n* = 1), Cochrane Oral Health’s Trials Register (*n* = 2), Cochrane Central Register of Controlled Trials (CENTRAL; *n* = 6), Cochrane Library (*n* = 1), Database of Open Access Journals (*n* = 1), Embase (*n* = 5), Google Scholar (*n* = 2), IndMed (India; *n* = 1), LILACS (*n* = 1), MEDLINE (*n* = 7), Open-SIGLE (*n* = 1), PubMed (*n* = 1), Sabinet (Africa; *n* = 1), ClinicalTrials.gov (*n* = 4), and World Health Organization International Clinical Trials Registry Platform (*n* = 3). The number of databases screened per systematic review ranged from 2 (Mejàre et al. 2003) to 8 (Mickenautsch and Yengopal 2016). Additional searches were performed on databases of dental journals (Li et al. 2020), dental journals were hand searched (Mickenautsch and Yengopal 2016), and reference lists were screened (Ahovuo-Saloranta et al. 2017; Li et al. 2020; Mejàre et al. 2003; Mickenautsch and Yengopal 2016; Ramamurthy et al. 2022; Rashed et al. 2022; Wright et al. 2016). The time frame of searches ranged from 1946 to 2021. Language restrictions were not applied in three systematic reviews (Ahovuo-Saloranta et al. 2017; Ramamurthy et al. 2022; Wright et al. 2016). For the other included systematic reviews, the language was restricted to English (Mickenautsch and Yengopal 2016), English and Chinese (Li et al. 2020), English and Arabic (Rashed et al. 2022), or to English, Swedish, Norwegian, Danish, German, French, Italian, Spanish (Mejàre et al. 2003).

**Table 1:** Study characteristics of the included systematic reviews on pit and fissure sealants in primary and permanent teeth of children and adolescents

No.	Author and year	No. and types of studies included	Participants	Type of teeth (no. of studies)	Intervention	Control	Outcomes assessed	Follow-up (months)	Quantitative synthesis performed (no. and type of studies)
1	Ahovuo-Saloranta et al. (2017)	38 RCTs (11 PG, 27 SM)	*Type of participants:* children and adolescents from general populations *Age:* 5–16 yrs	FPMs (*n* = 33) FPMs and SPMs (*n* = 4) SPMs (*n* = 1)	1. Resin-based (not 1st generation) or GI-based FS 2. Other/new type of FS	1. No FS 2. Resin-based FS (vs other type of FS); conventional FS (vs new type of FS)	• Caries incidence • DMF increment • Adverse events • Safety	12–108	Performed 17 RCTs (PG NR, SM NR) 9 RCTs (subgroup analyses)
2	Li et al. (2020)	8 RCTs (7 PG, 1 SM)	*Type of participants:* healthy children *Age:* 6–9 yrs	FPMs (*n* = 8)	1. Resin-based FS 2. GI-based FS 3. RMGI-based FS	1. FV (7700–22,600 ppm) 2. Water, blank, no application, OHE	• Caries incidence • DMFS increment	23–36	Performed 8 RCTs (7 PG, 1 SM)
3	Mejàre et al. (2003)	1 RCT (PG) 12 CCTs (3 PG, 8 SM, 1 NR)	*Type of participants:* children and adolescents *Age:* 5–14 yrs	Ps (*n* = 2) FPMs and SPMs (*n* = 13)	1. Resin-based FS (auto-/light-polymerized) 2. GI-based FS 3. RMGI-based FS	1. No treatment 2. Any other preventive treatment (only GI-based FS and xylitol chewing gum excluded)	• RR reduction • Prevented fraction • Net gain	24–60	Performed 8 non-RCTs
4	Mickenautsch and Yengopal (2016)	6 RCTs (4 PG, 1 SM, 1 partial SM)	*Type of participants:* children and adolescents *Age:* 5–11 yrs	PMs (*n* = 6)	HVGI-based FS (press-finger technique)	Resin-based FS	• No. of sealed teeth with caries from total no. of evaluated teeth	24–60	Performed 6 RCTs (4 PG, 1 SM, 1 partial SM)
5	Ramamurthy et al. (2022)	9 RCTs (1 PG, 8 SM)	*Type of participants:* children of general populations (2 RCTs with children from high-caries areas/at high caries risk) *Age:* 1.5–8 yrs	fpms and spms (*n* = 9)	FS	1. No FS 2. Any other FS material	• Caries incidence • Caries progression • Caries increment • Retention • Adverse events • Safety	12–30	Not performed
6	Rashed et al. (2022)	4 RCTs (2 PG, 2 NR)	*Type of participants:* schoolchildren *Age:* 6–9.1 yrs	FPMs (*n* = 4)	Resin-based FS	FV	• Caries incidence • DMFS increment	24	Performed Caries incidence: 3 RCTs (2 PG/1 NR) DMFS increment: 2 RCTs (1 PG/1 NR)
7	Wright et al. (2016)	23 RCTs (9 PG, 14 SM)	*Type of participants:* children and adolescents from general populations *Age:* 3–16 yrs	pms (*n* = 1) PMs (*n* = 23)	1. Resin-based FS (subcategory PMRC-based FS) 2. GI-based cement/FS (subcategory RMGI-based FS)	1. Any type of FS 2. No FS 3. FV	• Caries incidence • Retention • Adverse events	24– ≥ 84	Performed 22 RCTs (9 PG/13 SM)

Abbreviations:* CCTs* controlled clinical trials, *DMF(S)* decayed/missing/filled (surfaces), *fpms* first primary molars, *FPMs* first permanent molars, *FS* fissure sealant, *FV* fluoride varnish, *GI* glass-ionomer, *HVGI* high-viscosity glass-ionomer, *No* number, *NR* not reported, *OHE* oral health education, *PG* parallel group, *PMRC* polyacid-modified resin composite, *pms* primary molars, *PMs* permanent molars, *Ps* premolars, *RCTs* randomized clinical trials, *RMGI* resin-modified glass-ionomer, *SM* split-mouth, *spms* second primary molars, *SPMs* second permanent molars, *RR* relative risk, *yrs* years

**Table 2:** Results for caries incidence of included systematic reviews on sealants in primary and permanent teeth of children and adolescents

No.	Author and year	No. of participants, no. of teeth assessed baseline/latest follow-up (no. of studies)	Caries incidence (sealant material, no. of studies)
1	Ahovuo-Saloranta et al. (2017)	Participants: 7924 Teeth: 17,633 (*n* = 33) No. of teeth baseline NR (*n* = 5)	*Resin-based FS vs no FS:* 12 mos: OR 0.17; 95% CI [0.1, 0.3]; *I*^2^ = 80.79%; *p* < 0.0001 (Random) 24 mos: OR 0.12; 95% CI [0.08, 0.19];* I*^2^ = 72.51%; *p* < 0.0001 (Random) 36 mos: OR 0.17; 95% CI [0.11, 0.27]; *I*^2^ = 89.7%; *p* < 0.0001 (Random) 48 mos (*n* = 1): RR 0.24; 95% CI [0.12, 0.45]; *p* < 0.0001 (Fixed) 48–54 mos: OR 0.21; 95% CI [0.16, 0.28]; *I*^2^ = 44.99%; *p* < 0.0001 (Random) 60 mos: OR 0.31; 95% CI [0.23, 0.43]; *p* < 0.0001 (Fixed) 72 mos: RR 0.45; 95% CI [0.36, 0.58]; *p* < 0.0001 (Fixed) 84 mos: RR 0.45; 95% CI [0.34, 0.59]; *p* < 0.0001 (Fixed) 108 mos: RR 0.35; 95% CI [0.22, 0.55]; *p* < 0.0001 (Fixed) *GI-based FS vs no FS:* 24 mos (*n* = 1): OR 0.46; 95% CI [0.23, 0.91]; *p* = 0.03 (Fixed) *GI-based FS vs resin-based FS:* 12 mos (GIC): OR 1.47; 95% CI [0.64, 3.37]; *I*^2^ = 0%; *p* = 0.37 (Fixed) 12 mos (LVGIC): OR 1.56; 95% CI [0.63, 3.87]; *I*^2^ = 28.38%; *p* = 0.34 (Fixed) 12 mos (RMGIC): OR 1.06; 95% CI [0.13, 8.58]; *I*^2^ = 0%; *p* = 0.96 (Fixed) 24 mos (LVGIC): OR 1.67; 95% CI [0.87, 3.20]; *I*^2^ = 41.57%; *p* = 0.12 (Random) 24 mos (HVGIC): OR 1.36; 95% CI [0.56, 3.32]; *I*^2^ = 0%; *p* = 0.5 (Random) 24 mos (RMGIC): OR 2.92; 95% CI [1.77, 4.81]; *I*^2^ = 0%; *p* < 0.0001 (Random) 36–48 mos (LVGIC): OR 0.0; 95% CI [0.0, 0.0] 36–48 mos (RMGIC): OR 0.0; 95% CI [0.0, 0.0] 60 mos (HVGIC; *n* = 1): RR 0.38; 95% CI [0.09, 1.6]; *p* = 0.19 (Fixed) 84 mos (GIC; *n* = 1): RR 1.44; 95% CI [0.88, 2.35]; *p* = 0.15 (Fixed)
2	Li et al. (2020)	Participants: 3289 Teeth: 6878	*FV vs FS (enrolled children):* 24–36 mos: RR 1.12; 95% CI [0.60, 2.09]; *p* = 0.72; Chi^2^ = 2.45; *I*^2^ = 59% (Random) *FV vs FS (FPMs):* 24–36 mos: RR 1.29; 95% CI [0.95, 1.75]; *p* = 0.10; Chi^2^ = 20.85; *I*^2^ = 76% (Random) *FV vs FS (FPMs’ occlusal surfaces):* 24–36 mos: RR 1.33; 95% CI [0.83, 2.11]; *p* = 0.23; Chi^2^ = 20.49; *I*^2^ = 85% (Random)
3	Mejàre et al. (2003)	Participants: 3897 (*n* = 13) Teeth: 5984 (*n* = 11), two studies NR	*RR reduction:* Single application: 4–54% Repeated application: 69–93% *RR of developing caries:* RR 0.67; 95% CI [0.55, 0.83]; *p* < 0.001 (Random); corresponding to 33% RR reduction
4	Mickenautsch and Yengopal (2016)	Participants: NR Teeth: 1909 baseline/1742 at 24 mos 567 baseline/429 at 36 mos 452 baseline/247 at 60 mos	*HVGIC vs resin-based FS:* 24 mos: RR 1.36; 95% CI [0.66, 2.78]; *I*^2^ = 24.2%; *p* = 0.4 (Random) 36 mos: RR 0.9; 95% CI [0.49, 1.67]; *I*^2^ = 2.0%; *p* = 0.75 (Random) 48 mos: RR 0.62; 95% CI [0.31, 1.21]; *I*^2^ = 0%; *p* = 0.16 (Random) 60 mos: RR 0.29; 95% CI [0.09, 0.95]; *I*^2^ = 0%; *p* = 0.04 (Random)
5	Ramamurthy et al. (2022)	Participants: 1120 Tooth surfaces: 1977	*Fluoride-releasing resin-based FSs vs no FS (n = 1):* 12 mos: BB OR 1.21; 95% CI [0.37, 3.94] (Fixed) 24 mos: BB OR 0.76; 95% CI [0.41, 1.42] (Fixed) *GI-based FS vs no FS (n = 2; data not pooled):* 12 mos: OR 0.033; 95% CI [0.007, 0.149] (Fixed) 12–30 mos: OR 0.97; 95% CI [0.63, 1.49] (Fixed) *Autopolymerized FS vs light-polymerized resin-based FS (n = 1):* 24–36 mos: OR 0.58; 95% CI [0.15, 2.19] (Fixed)
6	Rashed et al. (2022)	Participants: 1249 Teeth (caries incidence): NR baseline/2622 latest follow-up Teeth (DMFS increment): NR baseline/1605 latest follow-up	*Resin-based FS vs FV:* 24 mos: RR 0.65; 95% CI [0.31, 1.38*]; *I*^2^ = 89%; *p* = 0.26 (Random, *data from forest plot)
7	Wright et al. (2016)	Participants: NR Teeth: NR baseline/9349 first follow-up	*FS vs no FS:* 24–36 mos (*n* = 9): OR 0.24; 95% CI [0.19, 0.30]; *I*^2^ = 41%; *p* < 0.00001 (Random) 48–84 mos (*n* = 3): OR 0.21; 95% CI [0.10, 0.44]; *I*^2^ = 77%; *p* < 0.0001 (Random) ≥ 84 mos (*n* = 2): OR 0.15; 95% CI [0.08, 0.27]; *I*^2^ = 50%; *p* < 0.00001 (Random) *FS vs FV:* 24–36 mos (*n* = 3): OR 0.27; 95% CI [0.11, 0.69]; *I*^2^ = 88%;* p* = 0.006 (Random) 48–84 mos (*n* = 2): OR 0.19; 95% CI [0.07, 0.51]; *I*^2^ = 80%; *p* = 0.0008 (Random) ≥ 84 mos (*n* = 1): OR 0.29; 95% CI [0.17, 0.49]; *p* < 0.00001 (Random) *GI-based FS vs resin-based FS:* 24–36 mos (*n* = 10): OR 0.71; 95% CI [0.32, 1.57]; *I*^2^ = 81%; *p* = 0.39 (Random) 48–84 mos (*n* = 2): OR 0.37; 95% CI [0.14, 1.00]; *I*^2^ = 0%; *p* = 0.05 (Random) *GI-based FS vs RMGI-based FS:* 24–36 mos (*n* = 1): OR 1.41; 95% CI [0.65, 3.07]; *p* = 0.38 (Random) *RMGI-based FS vs PMRC-based FS:* 24–36 mos (*n* = 1): OR 0.44; 95% CI [0.11, 1.82];* p* = 0.26 (Random) *PMRC-based FS vs resin-based FS:* 24–36 mos (*n* = 2): OR 1.01; 95% CI [0.48, 2.14], *I*^2^ = 0%; *p* = 0.97 (Random)

Abbreviations: *BB OR* Becker Balagtas odds ratio, *DMF(S)* decayed/missing/filled (surfaces), *FPMs* first permanent molars, *FS* fissure sealant, *FV* fluoride varnish, *GI(C)* glass-ionomer (cement), *HVGI(C)* high-viscosity glass-ionomer cement, *LVGI(C)* low-viscosity glass-ionomer cement, *mos* months, *No* number, *NR* not reported, *OR* odds ratio, *PMRC* polyacid-modified resin composite, *RMGI(C)* resin-modified glass-ionomer (cement), *RR* relative risk, *vs* versus

**Table 3:** Results for caries DMFS increment, retention rate, adverse events, and conclusions according to the review authors for the comparisons assessed among the included systematic reviews on sealants in primary and permanent teeth of children and adolescents

No.	Author and year	Comparisons assessed	DMFS increment (no. of studies)	Retention rate (no. of studies)	Adverse events	Conclusions according to the review authors
1	Ahovuo-Saloranta et al. (2017)	Resin-based FS vs no FS GI-based FS vs no FS GI-based FS vs resin-based FS	*Resin-based FS vs no FS:* DFS increment at 24 mos (*n* = 1): MD −0.65; 95% CI [−0.83, −0.47]; *p* < 0.0001 (Fixed) DMFS increment at 24 mos (*n* = 1): MD −0.24; 95% CI [−0.36, −0.12]; *p* < 0.0001 (Fixed) *GI-based FS vs no FS:* 24 mos (*n* = 1): MD −0.18; 95% CI [−0.39, 0.03]; *p* = 0.09 (Fixed) *GI-based FS vs resin-based FS:* 24 mos (*n* = 1): MD 0.47; 95% CI [0.31, 0.63]; *p* < 0.0001 (Fixed)	*Complete retention of resin-based FS vs no FS:* 12 mos: 53% (*n* = 1) to 90% (*n* = 4) 24 mos (*n* = 7): > 80% 36 mos: 41% (*n* = 1) to 87% (*n* = 1) 48–54 mos (*n* = 3): 70% 108 mos (*n* = 1): 39% 7% FS loss (*n* = 1, follow-up NR) *GI-based FS:* 24 mos (*n* = 1): < 1% complete retention 35% FS loss (*n* = 1, follow-up NR) *LVGI-based FS vs resin-based FS:* Better retention for resin-based FS (*n* = 8) 36–48 mos (*n* = 5): 8% vs 76% complete retention *HVGI-based FS vs resin-based FS:* Inconclusive results (*n* = 3) 24 mos (*n* = 1): 20% HVGI-based FS loss vs 14% resin-based FS loss 24 mos (*n* = 1): 55–79% complete or partial retention for HVGI-based FS 60 mos (*n* = 1): 58% vs 42% complete or partial retention *RMGI-based FS vs no FS:* 24 mos (*n* = 1): 16% FS loss *RMGI-based FS vs resin-based FS:* Better complete retention for resin-based FS (36 mos: 5% vs 94%)	Four trials examining adverse events did not report any	• Caries reduction for resin-based FS on permanent molars’ occlusal surfaces when compared with non-use of FS • Caries incidence 16–70% in controls at 24 mos → absolute caries risk reduction 11–51% (quality of evidence: moderate); similar outcome at ≤ 48-month follow-up. FS effective at follow-up > 48 mos, but limited evidence • Adverse events: limited information, no adverse events mentioned in studies reporting on it • Insufficient evidence for effectiveness of GI-based FSs • Insufficient evidence relative effectiveness of different FS materials in head-to-head comparisons • Further RCTs on clinical effectiveness of FSs needed
2	Li et al. (2020)	Resin-based FS vs FV Resin-based FS vs FV vs water Resin-based FS vs FV vs blank RMGI-based FS vs FV vs OHE GI-based FS vs FV vs blank	*FV vs FS (FPMs):* 24–36 mos: MD 0.13; 95% CI [−0.09, 0.34]; *p* = 0.25; Chi^2^ = 19.54; *I*^2^ = 85% (Random)	NR	NR	• Biannual FV application not significantly different caries preventive effect compared with FS at 24–36 mos • Treatment choice between both may be based on other factors, such as technique sensitivity, local availability, and treatment costs
3	Mejàre et al. (2003)	Autopolymerized resin-based FS vs NR Autopolymerized resin-based FS vs GI-based FS Light-polymerized resin-based FS vs NR RMGI-based FS vs GI-based FS	NR	NR	NR	• Caries-preventive effect of resin-based FSs applied in FPMs (limited evidence) • Insufficient evidence for effectiveness of FSs in primary molars, premolars, second permanent molars, and populations at low/high caries risk • Insufficient evidence for caries-preventive effect of GI-based FSs
4	Mickenautsch and Yengopal (2016)	HVGI-based FS vs resin-based FS	NR	NR	NR	• HVGI-based FSs not less caries-preventive than resin-based FS on occlusal surfaces of completely erupted permanent molars according to available evidence • Comparable caries-preventive effect between both after 48 mos • Poor evidence for any superiority of HVGI-based FSs compared with resin-based FSs after 60 mos • High RoB among included RCTs impairs validity of results • Future research on this question needed
5	Ramamurthy et al. (2022)	Fluoride-releasing resin-based FS vs no FS GI-based FS vs no FS GI-based FS vs resin-based FS Fluoride-releasing resin-based FS vs resin-based FS Flowable composite vs Fluoride-releasing resin-based FS Autopolymerised FS vs light polymerized resin-based FS	NR	*GI-based FS vs fluoride-releasing resin-based FS:* 24 mos (*n* = 1): BB OR 0.20; 95% CI [0.11, 0.36] (Fixed) *Auto- vs light-polymerized resin-based FS:* 24–36 mos (*n* = 1): OR 0.68; 95% CI [0.33, 1.44] (Fixed)	Uncomfortable feeling with strong gag reflex (1 child), uncomfortable feeling (8 children)	• Very low to low certainty of the evidence for the comparisons assessed in primary dentition • Small RCTs with limited number of events included • Most studies of split-mouth design with known limitations (e.g., analysis, reporting). Few information about adverse events • Need for future robust RCTs on caries prevention and retention rate of FSs in primary molars
6	Rashed et al. (2022)	Resin-based FS vs FV	*Resin-based FS vs FV:* 24 mos: MD −0.13; 95% CI [−0.67, 0.40]; Chi^2^ = 12.74, *I*^2^ = 92%; *p* = 0.63 (Random)	NR	NR	• No significant difference between FS and FV regarding caries-preventive effect after 24 mos, both seem to have a caries-preventive effect • FV less expensive and simpler application • Future well-conducted RCTs needed
7	Wright et al. (2016)	FS vs no FS FS vs FV GI-based FS vs resin-based FS GI-based FS vs RMGI-based FS RMGI-based FS vs PMRC-based FS PMRC-based FS vs resin-based FS	NR	*GI-based FS vs resin-based FS:* 24–36 mos (*n* = 10): OR 5.06; 95% CI [1.81, 14.13]; *I*^2^ = 96%; *p* = 0.002 (Random) 48–84 mos (*n* = 2): OR 2.08; 95% CI [0.15, 27.95]; *I*^2^ = 89%; *p* = 0.58 (Random) *GI-based FS vs RMGI-based FS:* 24–36 mos (*n* = 1): OR 3.21; 95% CI [1.87, 5.51]; *p* < 0.0001 (Random) *RMGI-based FS vs PMRC-based FS:* 24–36 mos (*n* = 1): OR 1.17; 95% CI [0.52, 2.66]; *p* = 0.7 (Random) *PMRC-based FS vs resin-based FS:* 24–36 mos (*n* = 2): OR 0.87; 95% CI [0.12, 6.21]; *I*^2^ = 81%; *p* = 0.89 (Random)	No adverse events reported (2 RCTs)	• About 80% reduction in caries incidence on permanent molars’ occlusal surfaces in children and adolescents for FS compared with non-use of FS (quality of evidence: moderate) • About 70% reduction in caries incidence on permanent molars’ occlusal surfaces in children and adolescents for FS compared with FV (quality of evidence: low) • FS with proven superiority in caries prevention and caries arrest of non-cavitated carious lesions compared with non-use of FSs/FV • No adverse events reported • Impossible to rank FSs based on their effectiveness assessed in studies on sealant material comparisons

Abbreviations:* FPMs* first permanent molars, *FS(s)* fissure sealant(s), *FV* fluoride varnish, *GI* glass-ionomer, *HVGI(C)* high-viscosity glass-ionomer (cement), *LVGI* low-viscosity glass-ionomer, *MD* mean difference, *mos* months, *No* number, *NR* not reported, *OHE* oral health education, *OR* odds ratio, *PMRC* polyacid-modified resin-based, *RCTs* randomized controlled trials, *RMGI* resin-modified glass-ionomer, *RoB* risk of bias, *vs* versus

A sum of 89 randomized controlled clinical trials (RCTs) was included in the systematic reviews, among them 35 were in parallel group design, 51 in split-mouth design, 1 in partial split mouth design, and for 2 RCTs the study design was not specified. In one systematic review, the study design was not restricted to RCTs and controlled clinical trials were also accepted (Mejàre et al. 2003).

The age span of included children and/or adolescents was between 1.5 and 16 years. Permanent molars were included in 92 primary studies, two primary studies further reported on the inclusion of permanent premolars, and primary molars were sealed in 10 primary studies (Table 1).

The caries prevalence of populations under investigation was reported in three included systematic reviews (Ahovuo-Saloranta et al. 2017, Mickenautsch and Yengopal 2016; Ramamurthy et al. 2022). Regarding the extent of caries being permitted for teeth to be sealed, 6 studies included sound teeth (Ahovuo-Saloranta et al. 2017; Li et al. 2020; Mickenautsch and Yengopal 2016; Ramamurthy et al. 2022; Rashed et al. 2022; Wright et al. 2016) and 4 studies teeth with initial carious lesions (Ahovuo-Saloranta et al. 2017; Li et al. 2020; Ramamurthy et al. 2022; Wright et al. 2016), whereas no information about the caries status of included teeth were provided in the remaining study (Mejàre et al. 2003).

There were few information about pretreatments and the type of isolation provided, even though a wide range of interventions was assessed among the included systematic reviews. Four systematic reviews compared the use of different sealant materials with the non-use of sealants (Ahovuo-Saloranta et al. 2017; Mejàre et al. 2003; Ramamurthy et al. 2022; Wright et al. 2016). Sealants used for this comparison were either sealants in general (Wright et al. 2016), resin-based sealants or GI-based sealants including various GIC subtypes (Ahovuo-Saloranta et al. 2017; Mejàre et al. 2003), and fluoride-releasing resin-based sealants or glass ionomer (GI)-based sealant (Ramamurthy et al. 2022).

Three systematic reviews assessed the comparison of sealants and fluoride varnish (Li et al. 2020; Rashed et al. 2022; Wright et al. 2016). In one systematic review, sealants in general were compared to fluoride varnish application (Wright et al. 2016). Li et al. (2020) specified the sealant materials included as resin-based sealants, resin-modified glass ionomer (RMGI)-based sealants, and GI-based sealants, which were compared to fluoride varnish application and further (negative) control groups (Li et al. 2020). The other systematic review included resin-based sealants as intervention being compared to fluoride varnish application (Rashed et al. 2022).

The following direct comparisons of different sealant materials were further assessed:

- GI-based sealants vs RMGI-based sealants (Mejàre et al. 2003; Wright et al. 2016),
- RMGI-based sealants vs polyacid-modified resin-based sealants (Wright et al. 2016),
- polyacid-modified resin-based sealants vs resin-based sealants (Wright et al. 2016),
- GI-based sealants (including subtypes) vs resin-based sealants (Ahovuo-Saloranta et al. 2017; Mejàre et al. 2003; Mickenautsch and Yengopal 2016; Ramamurthy et al. 2022; Wright et al. 2016),
- fluoride-releasing resin-based sealants vs resin-based sealants (Ramamurthy et al. 2022),
- fluoride-releasing resin-based sealants vs flowable composite resins (Ramamurthy et al. 2022), and
- autopolymerised sealants vs light-polymerised resin-based sealants (Ramamurthy et al. 2022).

In one systematic review, the comparisons were not clearly specified for all included studies (Mejàre et al. 2003). One systematic review included primary studies, in which high-viscosity glass-ionomer cements (HVGICs) applied by press-finger technique were compared to the conventional application of resin-based sealants (Mickenautsch and Yengopal 2016).

Outcomes assessed were caries incidence (Ahovuo-Saloranta et al. 2017; Li et al. 2020; Mickenautsch and Yengopal 2016; Ramamurthy et al. 2022; Rashed et al. 2022; Wright et al. 2016), caries progression (Ramamurthy et al. 2022), dmft/s or DMFT/S increment (Ahovuo-Saloranta et al. 2017; Li et al. 2020; Ramamurthy et al. 2022; Rashed et al. 2022), retention rate (Ramamurthy et al. 2022; Wright et al. 2016), relative risk reduction (Mejàre et al. 2003), prevented fraction (Mejàre et al. 2003), and net gain (Mejàre et al. 2003). In addition, adverse events were reported as secondary outcomes in three systematic reviews (Ahovuo-Saloranta et al. 2017; Ramamurthy et al. 2022; Wright et al. 2016).

The follow-up for pit and fissure sealings evaluated in included systematic reviews ranged from 12 to 108 months. Meta-analyses were performed in six included systematic reviews (Ahovuo-Saloranta et al. 2017; Li et al. 2020; Mejàre et al. 2003; Mickenautsch and Yengopal 2016; Rashed et al. 2022; Wright et al. 2016).

### Risk of bias assessment

For the risk of bias assessment (Table 4), 5 included systematic reviews used the Cochrane Risk of Bias tool (Ahovuo-Saloranta et al. 2017; Li et al. 2020; Ramamurthy et al. 2022; Rashed et al. 2022; Wright et al. 2016) and 2 had own criteria (Mejàre et al. 2003; Mickenautsch and Yengopal 2016). The overall risk of bias in included systematic reviews was rated as low in one (Ramamurthy et al. 2022) and as high in the remaining six systematic reviews (Ahovuo-Saloranta et al. 2017; Li et al. 2020; Mejàre et al. 2003; Mickenautsch and Yengopal 2016; Rashed et al. 2022; Wright et al. 2016). In three systematic reviews, concerns were raised regarding the identification and selection due to language restrictions applied (Li et al. 2020; Mejàre et al. 2003; Mickenautsch and Yengopal 2016) and in one due to the restriction in years of publication of included studies (Mejàre et al. 2003). In one systematic review, information about the number of high-risk studies included was unclear due to differences between the data in the text and in the tables (Li et al. 2020). Two systematic reviews applied own criteria for the risk of bias assessment, which resulted in unclear concerns about the study appraisal (Mejàre et al. 2003; Mickenautsch and Yengopal 2016). For domain 4 “*synthesis and findings*”, the fact that 6 systematic reviews performed meta-analyses by including primary studies at an unclear and/or high risk of bias raised high concerns because it affects the quality of results and conclusions drawn (Ahovuo-Saloranta et al. 2017; Li et al. 2020; Mejàre et al. 2003; Mickenautsch and Yengopal 2016; Rashed et al. 2022; Wright et al. 2016).

**Table 4:** Risk of bias assessment of the included systematic reviews with risk of bias in systematic reviews (ROBIS) tool (Whiting et al. 2016)

Authors and year	Risk of bias assessment tool used	Phase 2				Phase 3	Comments
		1. Study eligibility criteria	2. Identification and selection of studies	3. Data collection and study appraisal	4. Synthesis and findings	Risk of bias in the review	
Ahovuo-Saloranta et al. (2017 )	Cochrane Risk of Bias Tool						Publication of pre-specified review protocol NR; exclusion of studies with an abstract only; studies with high RoB included in meta-analyses (high risk of bias for all studies regarding blinding of outcome assessment)
Li et al. (2020)	Cochrane Risk of Bias Tool						Language restrictions; studies with unclear and high risk of bias included in meta-analyses
Mejàre et al. (2003)	Own criteria						Restrictions in years of publication, not standardised method of risk of bias assessment, heterogeneity not addressed, studies with unclear and high risk of bias included in meta-analysis
Mickenautsch and Yengopal (2016)	Own criteria						Language restrictions; own RoB criteria assessing selection, detection, and performance bias with assumption of attrition bias used; studies with unclear and high RoB included in meta-analyses
Ramamurthy et al. (2022)	Cochrane Risk of Bias Tool						Publication of pre-specified review protocol NR, no meta-analysis performed
Rashed et al. (2022)	Cochrane Risk of Bias tool						Publication year restriction (1980–2022); studies with unclear and high RoB included in meta-analyses
Wright et al. (2016)	Cochrane Risk of Bias tool						Publication of pre-specified review protocol NR; publication year restriction (1971–2013, 2013–2016); studies with unclear and high RoB included in meta-analyses

Low concern

Unclear concern

High concern

In addition, three systematic reviews assessed the certainty of evidence using the Grading of Recommendations, Assessment, Development and Evaluation (GRADE) methodology (Ahovuo-Saloranta et al. 2017; Ramamurthy et al. 2022; Wright et al. 2016).

### Primary and secondary outcomes for primary molars

One systematic review without quantitative synthesis investigated the clinical effectiveness of sealants on 1977 tooth surfaces of primary molars in 1120 children aged 1.5 to 8 years, in which literature published until February 2021 was included (Ramamurthy et al. 2022).

When comparing sealants to the non-use of sealants in primary molars, heterogeneity among the three included primary studies did not allow for pooling data (Chabadel et al. 2021; Chadwick et al. 2005; Joshi et al. 2019). The review authors reported on insufficient evidence to detect a difference between fluoride-releasing sealants and the non-use of sealants regarding the caries incidence at the 24-month follow-up. For primary molars treated with GI-based sealants versus the non-use of sealants in primary molars, results were ambiguous for follow-ups ranging from 12 to 30 months. All in all, these results were rated as being of low quality of evidence. The comparison of different sealant materials in the same systematic review showed that the reported caries incidence was low for all sealants under investigation. Again, the heterogeneity of the six included primary studies (Baca et al. 2007; Corona et al. 2005; Ganesh and Tandon 2006; Hotuman et al. 1998; Ren et al. 2011; Unal et al. 2015) precluded from quantitative analysis and the certainty of evidence for these results was very low to low (Ramamurthy et al. 2022).

Concerning secondary outcomes, the same systematic review reported on one primary study mentioning a gag reflex and an uncomfortable feeling as adverse event (Ramamurthy et al. 2022; Ren et al. 2011).

### Caries incidence in permanent molars

#### Comparison of sealant versus non-use of sealant

Three systematic reviews reported on caries incidence, caries increment, and caries risk reduction for the comparison of sealants with no treatment in permanent molars of children and adolescents (Ahovuo-Saloranta et al. 2017; Mejàre et al. 2003; Wright et al. 2016).

One systematic review with meta-analysis summarized the results of 9 primary studies (Bojanini et al. 1976; Bravo et al. 1996; Erdogan and Alaçam 1987; Liu et al. 2012; Mertz-Fairhurst et al. 1984; Pereira et al. 2003; Richardson et al. 1980b; Splieth et al. 2001; Tagliaferro et al. 2011) including 3542 participants about the caries incidence on permanent molars’ occlusal surfaces and showed a significant caries risk reduction of 76% (OR 0.24; 95% CI [0.19, 0.30]; *I*^2^ = 41%; *p* < 0.00001) for sealed compared to unsealed surfaces after 24 to 36 months (Wright et al. 2016). Furthermore, participants with sealants had a reduction in the risk of caries incidence by 79% (3 studies; 752 participants; OR 0.21; 95% CI [0.10, 0.44]; *I*^2^ = 77%; *p* < 0.0001) after 48 to 84 months (Bravo et al. 1996; Erdogan and Alaçam 1987; Mertz-Fairhurst et al. 1984), and by 85% (2 studies; 446 participants; OR 0.15; 95% CI [0.08, 0.27]; *I*^2^ = 50%; *p* < 0.00001) after 84 or more months follow-up as compared to the non-use of sealants (Bravo et al. 1996; Mertz-Fairhurst et al. 1984). The review authors rated the quality of evidence to be moderate to low due to concerns regarding the risk of bias assessment and heterogeneity of included studies (Wright et al. 2016).

Ahovuo-Saloranta et al. (2017) assessed the caries incidence when either resin-based or GI-based sealants were compared with sealant non-use in first permanent molars of 5- to 13-year-old children (Ahovuo-Saloranta et al. 2017). For resin-based sealants of the second or later generations with follow-ups ranging from 12 to 54 months, the results of 7 primary studies each for the 12-, 24-, and 36-month follow-up (Bojanini et al. 1976; Brooks et al. 1979; Charbeneau and Dennison 1979; Erdogan and Alaçam 1987; Hunter 1988; Liu et al. 2012, 2014a, b; Muller-Bolla et al. 2013; Richardson et al. 1978; Rock et al. 1978; Sheykholeslam and Houpt 1978) and 4 primary studies with 48- to 54-month follow-up (Brooks et al. 1979; Charbeneau and Dennison 1979; Erdogan and Alaçam 1987; Richardson et al. 1978) were pooled and quantitatively analyzed. For all these follow-ups, meta-analyses showed highly significant results for the comparison between resin-based sealants and not treatment (*p* < 0.00001) meaning that resin-based sealants efficiently prevented caries in children’s first permanent molars. Whereas the quality of evidence was moderate at 24 months, the quantity and quality of evidence declined with longer follow-ups. For GI-based sealants, the review authors found inconclusive results, which were rated as being of very low quality of evidence for the 24-month follow-up (Ahovuo-Saloranta et al. 2017).

Another systematic review published in 2003 included 13 primary studies with a sum of 3897 participants comparing the caries increment between pit and fissure sealings on occlusal surfaces and no treatment or other caries preventive measures in children and adolescents aged up to 14 years at the beginning of the trial (Mejàre et al. 2003). The majority of clinical trials was conducted in the 1970s and a single application was performed in most of the cases showing a relative caries risk reduction of 4–54% (Charbeneau and Dennison 1979; Going et al. 1977; Higson 1976; Horowitz et al. 1977; Leake and Martinello 1976; Pereira et al. 2003; Poulsen et al. 2001; Raadal et al. 1984; Richardson et al. 1980b; Stephen 1978; Thylstrup and Poulsen 1978) as compared to repeated applications with 69–93% (Bravo et al. 1997a, b, c; Songpaisan et al. 1995). The results confirmed the relationship between caries risk reduction and complete sealant retention. When performing a meta-analysis with 8 primary studies of moderate to high risk of bias (Charbeneau and Dennison 1979; Going et al. 1977; Higson 1976; Horowitz et al. 1977; Leake and Martinello 1976; Raadal et al. 1984; Richardson et al. 1980b; Stephen 1978), the review authors calculated a significant relative caries risk reduction of 33% (RR 0.67; 95% CI [0.55, 0.83]; *p* < 0.001) for resin-based sealants applied to the occlusal surfaces of first permanent molars (Mejàre et al. 2003).

#### Comparison of sealant versus fluoride varnish

Three systematic reviews provided inconsistent data for the caries incidence when the use of sealants was compared to fluoride varnish application (Li et al. 2020; Rashed et al. 2022; Wright et al. 2016). Whereas one systematic review reported on improved caries reduction rates when sealants were applied on pits and fissures of permanents molars (Wright et al. 2016), two other reviews found no statistically significant difference for the caries incidence between sealants and fluoride varnishes (Li et al. 2020; Rashed et al. 2022).

For this comparison, Wright et al. (2016) estimated a reduction in caries incidence by 73% (3 primary studies with 1715 participants; OR 0.27; 95% CI [0.11, 0.69]; *I*^2^ = 88%; *p* = 0.006) at 24–36 months follow-up (Bravo et al. 1996; Houpt and Shey 1983; Liu et al. 2012), and by 81% (2 primary studies with 472 participants; OR 0.19; 95% CI [0.07, 0.51]; *I*^2^ = 80%;* p* = 0.0008) after 48–84 months (Bravo et al. 1996; Houpt and Shey 1983) when a resin-based sealant versus a fluoride varnish was applied on permanent molars (Wright et al. 2016). The quality of evidence was low for both follow-ups due to the heterogeneity and the increased risk of bias. The review authors calculated an OR of 0.29 (95% CI [0.17, 0.49]; *p* < 0.00001; one study; 242 participants) for follow-ups of 84 months or more (Bravo et al. 1996) providing low-quality of evidence for the reduction of caries incidence when pit and fissure sealings were compared with fluoride varnish application on permanent molars (Wright et al. 2016).

In the systematic review and meta-analysis published by Li et al. (2020), the quantitative analysis revealed that the caries incidence for the comparison of sealants (resin-based sealants, conventional GI-based sealants, and RMGI-based sealants) and fluoride varnish was without significant differences with regard to 1072 children enrolled (2 primary studies; RR 1.12; 95% CI [0.60, 2.09]; *I*^2^ = 59%; *p* = 0.72) (Chestnutt et al. 2017; Liu et al. 2012), 6878 first permanent molars (6 studies; RR 1.29; 95% CI [0.95, 1.75]; *I*^2^ = 76%; *p* = 0.10) (Bravo et al. 1996; Chestnutt et al. 2017; Ji et al. 2007; Liu et al. 2012; Raadal et al. 1984; Salem et al. 2014), and 6551 occlusal surfaces (4 primary studies; RR 1.33; 95% CI [0.83, 2.11]; *I*^2^ = 85%; *p* = 0.23) at the 24-to 36-month follow-up (Bravo et al. 1996; Chestnutt et al. 2017; Liu et al. 2012; Salem et al. 2014). Primary studies of unclear to high risk of bias were included in the meta-analyses and heterogeneity among them was increased (Li et al. 2020).

Rashed et al. (2022) included three primary studies of unclear to high risk of bias with 2622 first permanent molars in a meta-analysis which showed no significant difference for the caries increment (RR 0.65; 95% CI [0.31, 1.38]; *I*^2^ = 89%; *p* = 0.26) of sealed versus fluoridated first permanent molars in schoolchildren at the 24-month follow-up (Bravo et al. 1996, 1997a, b, c; Liu et al. 2012; Salem et al. 2014). In this systematic review, resin-based sealants were included while GI-based sealants were explicitly excluded (Rashed et al. 2022).

#### Comparison of sealant versus sealant

Three systematic reviews with meta-analyses provided information for the caries incidence when different sealant materials were compared with each other (Ahovuo-Saloranta et al. 2017; Mickenautsch and Yengopal 2016; Wright et al. 2016).

In one systematic review and meta-analysis, different GI-based sealants were compared with resin-based sealants for follow-ups between 12 and 84 months (Ahovuo-Saloranta et al. 2017). At the 12-month follow-up, the OR of GI-based sealants compared to resin-based sealants was 1.47 for GI-based sealants (95% CI [0.64, 3.37]; *I*^2^ = 0%; *p* = 0.37), 1.56 for low-viscosity GI-based sealants (4 studies; 95% CI [0.63, 3.87]; *I*^2^ = 28.38%; *p* = 0.34) (Dhar and Chen 2012; Karlzén-Reuterving and van Dijken 1995; Rock et al. 1996; Sipahier and Ulusu 1995), and 1.06 for resin-modified GI-based sealants (2 studies; 95% CI [0.13, 8.58]; *I*^2^ = 0%; *p* = 0.96) (Amin 2008; Baseggio et al. 2010). At the 24-month follow up, low-viscosity GI-based sealants versus resin-based sealants had an OR of 1.67 (10 studies; 95% CI [0.87, 3.20]; *I*^2^ = 41.57%; *p* = 0.12) (Antonson et al. 2012; Chen and Liu 2013; Dhar and Chen 2012; Forss and Halme 1998; Ganesh and Tandon 2006; Karlzén-Reuterving and van Dijken 1995; Mills and Ball 1993; Poulsen et al. 2001; Rock et al. 1996; Williams et al. 1996), high-viscosity GI-based sealants versus resin-based sealant had an OR of 1.36 (2 studies; 95% CI [0.56, 3.32]; *I*^2^ = 0%; *p* = 0.5) (Chen et al. 2012; Liu et al. 2014a, b), and resin-modified GI-based versus resin-based sealants had an OR of 2.92 (2 studies; 95% CI [1.77, 4.81]; *I*^2^ = 0%; *p* < 0.0001) (Amin 2008; Baseggio et al. 2010). For longer follow-ups, a qualitative synthesis was not performed due to substantial heterogeneity. Final statements about the relative effectiveness of various sealant materials could not be drawn by the review authors (Ahovuo-Saloranta et al. 2017).

At the 24- to 36-month follow-up, Wright et al. (2016) compared the results of 10 primary studies (Amin 2008; Antonson et al. 2012; Arrow and Riordan 1995; Baseggio et al. 2010; Chen et al. 2012; Chen and Liu 2013; Dhar and Chen 2012; Guler and Yilmaz 2013; Haznedaroglu et al. 2016; Pardi et al. 2005) for the caries incidence of GI-based sealants compared with resin-based sealants (OR 0.71; 95% CI [0.32, 1.57]; *I*^2^ = 81%). For the 4741 included participants, the caries risk reduction of 29% for GI-based versus resin-based sealings did not reach statistical significance (*p* = 0.39). For the same comparison at 48–84 months, the OR was 0.37 (2 studies; 145 participants; 95% CI [0.14, 1.00]; *I*^2^ = 0%; *p* = 0.05) (Barja-Fidalgo et al. 2009; Haznedaroglu et al. 2016). Further comparisons assessed at 24- to 36-months were: GI-based versus resin-modified GI-based sealants (1 study; OR 1.41; 95% CI [0.65, 3.07]; *p* = 0.38) (Pereira et al. 2003); resin-modified GI-based versus polyacid-modified resin-based sealants (1 primary study; OR 0.44; 95% CI [0.11, 1.82]; *p* = 0.26) (Pardi et al. 2005); polyacid-modified resin-based versus resin-based sealants (2 primary studies; OR 1.01; 95% CI [0.48, 2.14]; *I*^2^ = 0%;* p* = 0.97) (Güngör et al. 2004; Pardi et al. 2005).

The third systematic review included six primary studies (Barja-Fidalgo et al. 2009; Beiruti et al. 2006a, b; Chen et al. 2012; Hilgert et al. 2015; Liu et al. 2014a, b; Oba et al. 2009) comparing pit and fissure sealings with HVGIC applied by press-finger technique versus the conventional application of resin-based sealants and found no significant differences for both for follow-ups up to 48 months “[…] *and borderline significant differences in favour of HVGIC sealants after 60 months (RR 0.29; 95% CI: 0.09–0.95; p* = *0.04/RD -0.07; 95% CI: -0.14, -0.01)* […]” (Mickenautsch and Yengopal 2016).

### DMFS increment in permanent molars

#### Comparison of sealant versus non-use of sealant

Ahovuo-Saloranta et al. (2017) provided information about the DMFS increment in permanent molars, when either resin-based sealants or GI-based sealants were compared with the non-use of sealants at the 24-month follow-up (Ahovuo-Saloranta et al. 2017). For resin-based sealants, the mean difference amounted to −0.65 (1 study; 95% CI [−0.83, −0.47]; *p* < 0.0001) for the DFS increment (Songpaisan et al. 1995), and to −0.24 (1 study; 95% CI [−0.36, −0.12]; *p* < 0.0001) for the DMFS increment (Tang et al. 2014). For the former, the mean difference of GI-based sealants versus no sealant was −0.18 (1 study; 95% CI [−0.39, 0.03];* p* = 0.09) (Songpaisan et al. 1995) at the same follow-up (Ahovuo-Saloranta et al. 2017).

#### Comparison of sealant versus fluoride varnish

When fluoride varnish was compared to sealants in general, the mean difference for the DMFS increment on occlusal surfaces was 0.13 (95% CI [−0.09, 0.34]; *I*^2^ = 85%; *p* = 0.25) after 24–36 months (Li et al. 2020). If sealants were compared to fluoride varnish application at a follow-up of 24 months, there was also no statically significant difference observed between both groups in another systematic review and meta-analysis (MD −0.13; 95% CI [−0.67, 0.40]; *I*^2^ = 92%; *p* = 0.63) (Rashed et al. 2022).

### Retention rate of sealants on permanent molars

One included systematic review provided data about the retention rate of different sealant materials being compared with each other at follow-ups of 24–36 months (14 comparisons) and 48–84 months (2 comparisons) (Wright et al. 2016). For the comparison between GI-based and resin-based sealants, there was an increased chance of sealant loss (108–406%) after 24–36 months (10 studies; 4741 participants; OR 5.06; 95% CI [1.81, 14.13]; *I*^2^ = 96%; *p* = 0.002) (Amin 2008; Antonson et al. 2012; Arrow and Riordan 1995; Baseggio et al. 2010; Chen et al. 2012; Chen and Liu 2013; Dhar and Chen 2012; Guler and Yilmaz 2013; Haznedaroglu et al. 2016; Pardi et al. 2005) and after 48–84 months (2 studies; 145 participants; OR 2.08; 95% CI [0.15, 27.95]; *I*^2^ = 89%; p = 0.58) (Barja-Fidalgo et al. 2009; Haznedaroglu et al. 2016) if GI-based sealants were used. When the latter were compared with resin-modified GI-based sealants, one primary study with 344 participants showed that the probability of retention loss was three times higher for GI-based sealants (OR 3.21; 95% CI [1.87, 5.51]; *p* < 0.0001) at the 24- to 36-month follow-up (Pereira et al. 2003). For the same follow-up, resin-modified GI-based sealants were more susceptible to sealant loss than polyacid-modified resin-based sealants (one study; 186 participants; OR 1.17; 95% CI [0.52, 2.66]; *p* = 0.7) (Pardi et al. 2005), and polyacid-modified resin-based sealants tended to have a reduced risk of sealant loss in comparison with resin-based sealants (2 studies; 322 participants; OR 0.87; 95% CI [0.12, 6.21]; *I*^2^ = 81%; *p* = 0.89) (Güngör et al. 2004; Pardi et al. 2005). However, the results for both comparisons did not reach statistical significance (*p* > 0.05). All in all, the review authors rated the quality of evidence as moderate to very low for the comparisons made due to the risk of bias and imprecision (Wright et al. 2016).

According to Ahovuo-Saloranta et al. (2017) resin-based sealants showed better complete retention rates than low-viscosity GI-based sealants (76% vs 8% at 36–48 months) and resin-modified GI-based sealants (94% vs 5% at 36 months), while results were inconclusive for the comparison with high-viscosity GI-based sealants. In general, complete retention of resin-based sealants varied between 53–90% (12-month follow-up), > 80% (24-month follow-up), 41–87% (36-month follow-up), 70% (48–54-month follow-up), and 39% (108-month follow-up).

**Fig. 2:**
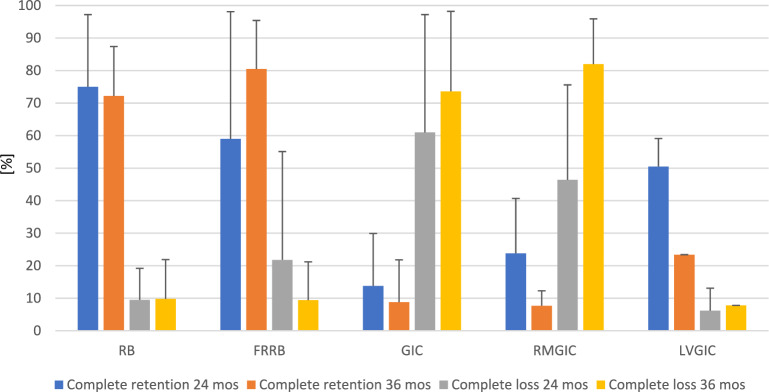
Mean percentage and standard deviation of complete sealant retention and complete sealant loss on permanent teeth after 24 and 36 months according to two included systematic reviews (Ahovuo-Saloranta et al. 2017; Wright et al. 2016). Abbreviations: *FRRB* fluoride-releasing resin-based sealant, *GIC* conventional glass-ionomer cement; *LVGIC* low-viscosity glass-ionomer cement, *RB* resin-based sealant, *RMGIC* resin-modified glass-ionomer cement

Figure 2 summarizes the retention rates of different sealant materials after 24–36 months. In general, resin-based and fluoride-releasing resin-based sealants showed higher rates of complete sealant retention and lower rates of complete sealant loss in comparison to conventional, resin-modified, and low-viscosity GI-based sealants.

### Secondary outcomes for permanent molars

Two included systematic reviews provided information about the secondary outcomes of this umbrella review (Ahovuo-Saloranta et al. 2017; Wright et al. 2016). Four primary studies (Bravo et al. 2005; Liu et al. 2012, 2014a, b; Muller-Bolla et al. 2013; Tagliaferro et al. 2011) of one systematic review included an assessment of adverse events related to pit and fissure sealings in permanent molars and none of them reported about the occurrence of adverse events (Ahovuo-Saloranta et al. 2017). Another systematic review included two primary studies (Bravo et al. 2005; Liu et al. 2012) including the assessment of adverse events and they also did not mention any (Wright et al. 2016).

### Quantitative synthesis of the results

Due to the substantial methodological and clinical heterogeneity and the high risk of bias of included systematic reviews, a quantitative synthesis of results was not deemed as appropriate for this umbrella review.

## Discussion

The rationale for conducting this study was that umbrella reviews, also known as overviews of reviews, summarize the available evidence published in separate systematic reviews by presenting or re-analyzing original outcome data followed by a critical appraisal (Higgins et al. 2019). In doing so, umbrella reviews allow for a comparison and contrast of data obtained from various interventions, thereby providing decision-makers with an expansive summary of the available evidence (Smith et al. 2011). This umbrella review adds to the existing literature by summarizing the results of different systematic reviews on the clinical effectiveness of pit and fissure sealants in one manuscript, which aimed to provide dental experts with information for the update of the EAPD guidelines on the use of pit and fissure sealants from 2004 (Welbury et al. 2004).

Based on the results of published systematic reviews, this umbrella review evaluated the clinical effectiveness of pit and fissure sealants applied on sound and initial carious primary molars and permanent (pre-)molars of children and adolescents over a follow-up of at least 12 months. It was chosen to include systematic reviews with a follow-up of at least 12 months for two reasons: (1) systematic reviews with a follow-up of at least 12 months were included in order not to be too restrictive in the systematic literature search process; (2) systematic reviews with longer follow-ups were included because they would be more likely to report differences in the clinical performance of sealant materials and caries lesion progression.

Seven systematic reviews citing a sum of 101 primary studies including double counting (Appendix 3), most of them RCTs, examined various sealant materials (different GI-based/polyacid-modified resin-based/different generations of resin-based sealants except for the first generation), chose a variety of comparisons (non-use of sealants, application of fluoride varnish, head-to-head material comparison), and outcomes (caries incidence, DMFS increment, retention rate). Regarding fluoride-releasing sealant materials (GI-based sealants or fluoride-releasing resin-based sealants), it has been shown in the literature that the fluoride-releasing properties are dependent on the material characteristics such as the fluoride and filler content or the matrices, which means that grouping different types of GICs (e.g., conventional GICs, RMGICs, HVGICs) under the umbrella term “*GI-based sealants*” may not consider possible material-specific differences in the fluoride release and uptake characteristics (Wiegand et al. 2007). Except for one systematic review investigating the press-finger technique for HVGIC sealants (Mickenautsch and Yengopal 2016), the other included systematic reviews reported on conventional sealant application.

According to Pieper et al. (2014a, b), a *CCA* of 0.074 (7.4%; moderate overlap) was calculated to interpret the overlap, since repetitive inclusion of primary studies in different systematic reviews may skew the real treatment effect (Pieper et al. 2014a, b). Regarding six publications, the review authors provided unequivocal decisions as to whether these publications were rated as different studies or as different reports of the same study. Ahovuo-Saloranta et al. (2017) grouped the four publications by Bravo and colleagues as reports of the same study (Bravo et al. 1996, 2005; Bravo et al. 1997a, b), while Li et al. (2020) and Rashed et al. (2022) included two of them as primary studies, and Mejàre et al. (2003) and Wright et al. (2016) included each of the publications (Ahovuo-Saloranta et al. 2017; Li et al. 2020; Mejàre et al. 2003; Rashed et al. 2022; Wright et al. 2016). As reported by Li et al. (2020), the final report of the ninth-year endpoint of this study was excluded from meta-analysis because the high drop-out rate (> 60%), the presence of sealed teeth in the fluoride varnish group (3.9%), and the termination of the biannual fluoride varnish application by the fourth year impaired the meaningfulness of the outcome (Li et al. 2020).

A similar case was observed for publications by Richardson and colleagues (Gibson and Richardson 1980; Gibson et al. 1982; Richardson et al. 1978, 1980a, b), for which one systematic review grouped five reports as belonging to one included primary study, and two other reviews included only one of these publications (Ahovuo-Saloranta et al. 2017; Mejàre et al. 2003; Wright et al. 2016). Since the publications by Bravo and colleagues could not be combined without changing the total number of included primary studies, we only grouped the publications by Richardson and colleagues to investigate the effect on the *CCA*, which slightly increased to 0.077 (7.7%; moderate overlap) after recalculation.

The primary studies included in the 7 systematic reviews (Appendix 3) were published between 1976 and 2021 covering a span of 45 years, while the included systematic reviews were published between 2003 and 2022. Especially one systematic review included several studies published in the 1970s (Mejàre et al. 2003). Apart from this systematic review, the other included ones were published between 2016 and 2022. The limitation arising from the inclusion of studies published earlier is that older generations of sealants with inferior performance compared to recently produced ones may have been investigated, which restricts the meaningfulness of results for the present situation. This may negatively influence the up-to-dateness, which has been shown to be rarely analyzed in umbrella reviews (Pieper et al. 2014a, b).

For the primary dentition, one systematic review including 1.5- to 8-year-old children showed no unequivocal superiority of fluoride-releasing resin-based and GI-based sealants in comparison to the non-use of sealants in primary molars up to the 30-month follow-up. When different sealant materials were compared with each other, the caries incidence was low for all sealant materials under investigation (Ramamurthy et al. 2022). The results of this umbrella review do not allow to draw best practice guidance about the use of sealants in primary molars due to the methodological heterogeneity of included primary studies (e.g., participants’ age, different sealant materials under investigation, varying follow-up periods) and the low to very low certainty of evidence presented in the systematic review (Ramamurthy et al. 2022).

For the permanent dentition, moderate to low quality of evidence was found for the clinical effectiveness of sealants in comparison to unsealed sound and initial carious occlusal surfaces of permanent molars in children and adolescents (Ahovuo-Saloranta et al. 2017; Wright et al. 2016). In comparison to unsealed permanent molars, the authors of one systematic review were “*moderately confident*” that resin-based sealants reduced caries over 48 months after the application, while no reliable conclusions could be drawn for GI-based sealants (Ahovuo-Saloranta et al. 2017).

For the comparison of sealants with fluoride varnish application, there was inconclusive evidence for the superiority of one treatment approach over the other for caries prevention in permanent molars of children based on three included systematic reviews evaluating this scenario (Li et al. 2020; Rashed et al. 2022; Wright et al. 2016). The sealant materials selected as comparator to the fluoride varnish application should be chosen carefully since the physical properties of the sealant material could influence the outcome. In that respect, the comparators chosen in the included systematic reviews were resin-based sealants (Li et al. 2020; Rashed et al. 2022; Wright et al. 2016), conventional GI-based sealants (Li et al. 2020), and RMGI-based sealants (Li et al. 2020). Li et al. (2020) found no statistically significant differences for the caries prevention in first permanent molars over a period of 24–36 months, when GI-based sealants or resin-based sealants were compared to biannual fluoride varnish application (Li et al. 2020). Based on these findings, the authors mentioned considering fluoride varnish application as caries-preventive measure especially in least developed and developing countries due to the lower costs and the easy handling (Li et al. 2020).

Another recently published systematic review and meta-analysis that did not meet the inclusion criteria of this umbrella review because combination treatment was included (application of sealants plus fluoride varnish) supported this finding (Kashbour et al. 2020). Based on insufficient available data and a very low certainty of evidence the review authors could not prove superiority of either resin-based sealants or fluoride varnishes, since both treatments seem to prevent caries in first permanent molars (Kashbour et al. 2020).

Available data from studies with head-to-head material comparisons provided insufficient evidence for a ranking of sealant materials regarding their caries prevention capabilities and retention rates (Wright et al. 2016). In addition, the relative effectiveness of resin-based sealants as compared to GI-based sealants was described as inconclusive in one systematic review (Ahovuo-Saloranta et al. 2017). The heterogeneity of clinical circumstances (among others the individual caries risk, the status of tooth eruption, the type of isolation, the cooperation of the child, the experience of the operator), the various comparisons between sealant materials made, and the paucity of long-term data may have precluded from making a final decision on which sealant material is the most beneficial. Wright et al. (2016) showed that the application of GI-based sealants was associated with a reduction in the risk of developing new caries lesions in comparison to resin-based sealants, however, this difference was not of statistical significance (*p* ≥ 0.05) (Wright et al. 2016). On the other hand, GI-based sealants were associated with a five times higher risk of retention loss after 24–36 months. The review authors could not find sufficient evidence for the effectiveness of these two sealant materials regarding the caries incidence level and the retention rate (Wright et al. 2016). In the clinical decision-making process, child-related factors (e.g., cooperation, caries risk) and tooth-related factors (e.g., status of tooth eruption, possibility of isolation) should be considered carefully in relation to the expected loss of retention when choosing a sealant material (Wright et al. 2016).

Across the three systematic reviews reporting on secondary outcomes of this umbrella review, serious adverse events associated with pit and fissure sealings were neither reported for primary teeth nor for permanent teeth in children and adolescents (Ahovuo-Saloranta et al. 2017; Ramamurthy et al. 2022; Wright et al. 2016).

### Strengths and limitations of the umbrella review

The main strength of this umbrella review was that the review process adhered to a predefined review protocol and the methodology followed the validated recommendations described in the Cochrane Handbook for Systematic Reviews of Interventions (Higgins et al. 2019) and the Preferred Reporting Items for Systematic Reviews and Meta-Analyses (PRISMA) (Page et al. 2021). The systematic literature search for this umbrella review was not restricted by publication year, which aimed at ensuring a high sensitivity of the search to minimize the risk of not retrieving systematic reviews relevant to this subject. Strict inclusion criteria were applied for the type (occlusal surfaces of primary molars or permanent premolars/molars) and the caries status of included teeth (either stated verbatim as sound teeth, respectively, teeth with initial carious lesions or referred to as ICDAS-II 0–3 (Ismail et al. 2007; Pitts 2004)), the age group (children and adolescents up to the age of 19 years), and the follow-up (at least 12 months). The rationale behind restricting the age of included participants was that this umbrella review aimed to evaluate the clinical effectiveness of sealants in children and adolescents. Studies with short follow-ups (< 12 months) were excluded because changes in the caries incidence or the DMFS increment need longer follow-ups, since the development of dentine caries takes time until it is to be detected (Albelasy et al. 2022; Askar et al. 2021; Hardie et al. 1977). Furthermore, a comprehensive spectrum of comparisons for both primary and permanent teeth was included due to more broadly defined outcome measures. Last but not least, no partial inclusion of review papers was accepted if the inclusion criteria were not met in all included clinical studies of the review and/or meta-analysis study.

Notwithstanding, the limitations of this umbrella review have to be mentioned as well, which are related to the heterogeneity of included systematic reviews and their extent of published data. Typically for various types of evidence synthesis, the results summarized in an umbrella review predominantly build upon the outcomes and the quality of the included systematic reviews, and finally of the primary studies included therein (Hartling et al. 2012). Heterogeneity was observed with respect to the study design of the included primary studies since not all systematic reviews included RCTs exclusively, and for RCTs studies in split-mouth design and parallel group design were included. Moreover, different outcomes were assessed, and the follow-up period was varying across the 7 included systematic reviews.

Only almost half of the included systematic reviews provided detailed information about the caries risk within the population, a fact that is of relevance when the caries incidence or the DMFS increment is the relevant outcome, since the treatment effect may vary between populations with low and high caries risk (Muller-Bolla et al. 2016).

Information about the setting, in which the treatment was conducted (e.g., university clinic, private dental practice, school setting), and the dependency on personnel sealing the teeth, such as dental students, experienced dentists, or auxiliary staff (Schill et al. 2022), was lacking. Further factors that were insufficiently reported in some of the included reviews, yet may impact the outcome, were the chosen type of pre-treatment (e.g., phosphoric etching time, adhesive application, preparation, other pre-treatments) (Bagherian and Shirazi 2016; Kucukyilmaz and Savas 2015), and the type of isolation (cotton rolls vs rubber dam) (Ganss et al. 1999).

Another issue was observed with regard to reporting about the outcome assessors and their blinded outcome assessment. It was stated by Ahovuo-Saloranta et al. (2017) that the risk of detection bias across the included primary studies was high because of the impossibility of blinding (Ahovuo-Saloranta et al. 2017). This decision seems reasonable, since blinding of outcome assessors is not feasible if materials of different appearance are used, or if sealings are compared either to no sealing or to fluoride varnish application. However, detection bias was rated heterogeneously among the included systematic reviews even though the comparisons mentioned above were made indicating a high risk of bias for this domain.

Ramamurthy et al. (2022) addressed the drawbacks of systematic reviews associated with the inclusion of small primary studies reporting on a restricted number of events, and the encounter of a heterogeneous study situation (Ramamurthy et al. 2022).

Finally, a quantitative synthesis of results was not feasible due to the increased risk of bias of included systematic reviews. Meta-analyses were performed in six of the systematic reviews by including primary studies of unclear and high risk of bias, which impairs the certainty of evidence of the results. As a matter of fact, a meta-analysis with systematic reviews having a high risk of bias regarding synthesis and finding was not performed in this umbrella review.

### Implications for future research

Based on the results of this umbrella review, a need for further rigorously planned and well conducted RCTs and systematic reviews on the clinical effectiveness of pit and fissure sealants compared either to each other or with the non-use of sealants in primary and permanent teeth of children and adolescents was observed (Mejàre et al. 2003; Ramamurthy et al. 2022; Rashed et al. 2022). It is recommendable to follow internationally accepted standards for trial reporting, such as the Consolidated Standards of Reporting Trials (CONSORT) statement (Ahovuo-Saloranta et al. 2017; Moher et al. 2010; Ramamurthy et al. 2022).

For primary studies to be included in systematic reviews, an adequate sample size estimation reduces the bias induced by including small RCTs with limited number of treatment effects (Ramamurthy et al. 2022). Moreover, proper randomization and allocation sequence concealment reduces the risk of “*bias arising from the randomization process*”, as described in the Cochrane Handbook for Systematic Reviews of Interventions (Higgins et al. 2019; Mickenautsch and Yengopal 2016).

Two types of study design are reported for RCTs, namely parallel group or split-mouth design, with the latter being frequently used in studies with paediatric participants. In split-mouth RCTs, each child has at least teeth located in different halves of the jaw being randomly assigned either to the intervention group or to the control group (Pozos-Guillén et al. 2017). During the follow-up, these teeth are exposed identical basic conditions, such as oral hygiene and nutrition, as they are located within the same oral cavity improving the RCTs’ power and accuracy by reducing the variability between participants (Pozos-Guillén et al. 2017; Zhu et al. 2017). The inclusion of fewer participants may be necessary to achieve a comparable power to parallel-group RCTs randomizing on mouth level (Zhu et al. 2017). However, recruitment may be hampered due to the fact that children with teeth of corresponding clinical condition are needed (e.g., proximal caries with comparable lesion depths for RCTs on the clinical effectiveness of restorations). Furthermore, the disadvantage of the so-called “carry-across effect” has to be considered carefully, meaning that there can be a cross-contamination of treatment effects (Pozos-Guillén et al. 2017). As far as fluoride varnish application is concerned, it was shown by Sköld-Larsson et al. (2000) that no significant carry-across effect in terms of increases in the fluoride concentration in plaque was observed if different fluoride varnishes were applied in the contralateral quadrant (Sköld-Larsson et al. 2000). The assumed dose-dependence of the carry-across effects combined with the small amounts of fluoride varnish applied can be found in literature as justification for the inclusion of studies in split-mouth design comparing sealants with fluoride varnish (Kashbour et al. 2020).

The inclusion criteria should clearly state the age of participants to be included, the caries risk of the population, and the accepted extent of carious lesions to be sealed (Ahovuo-Saloranta et al. 2017; Ramamurthy et al. 2022). Detailed demographic data about participants including the use of fluorides and further caries-preventive measures should be provided to allow for statistical analysis of confounding factors (Ahovuo-Saloranta et al. 2017; Ramamurthy et al. 2022). Information about the setting, the number and experience of calibrated operators, the type of pretreatment, the type of isolation, and the compliance of the child should be provided to allow for a better comparability of results. Studies conducted in populations at various caries risk (Mejàre et al. 2003) and caries prevalence levels can help show a more comprehensive picture of treatment effect as a function of caries risk/prevalence (Ahovuo-Saloranta et al. 2017).

Blinding of outcome assessors should be striven for to reduce the risk of detection bias, which may be impossible if sealant materials of different clinical appearance are used, and if sealants are compared to the non-use of sealants or fluoride varnish application (Ahovuo-Saloranta et al. 2017; Ramamurthy et al. 2022). Moreover, it should be clearly reported how sealant loss is dealt with meaning whether sealants are reapplied or not in case insufficient sealing, since this may have an impact on the outcome (Mejàre et al. 2003). The follow-up should be as long as reasonably possible to evaluate the long-term effect (Ahovuo-Saloranta et al. 2017; Mejàre et al. 2003; Rashed et al. 2022) with as little loss to follow-up of participants as possible, and drop-outs should be unequivocally stated.

Last but not least, studies with a low risk of bias should only be included in meta-analyses to improve the validity of results.

## Conclusions

In the frame of the present umbrella review, the following can be concluded:

- There is a lack of data to draw any solid conclusions on the clinical effectiveness of sealants for caries prevention in primary molars of children.
- There is a moderate quality of evidence that the application of sealants (especially resin-based sealants) on children’s permanent molars is more effective in preventing new caries lesions than leaving the teeth untreated.
- When resin-based and GI-based sealants are compared with fluoride varnish, there is insufficient evidence for the superiority of one treatment modality over the other for caries prevention in permanent molars of children.
- Based on the available data, it was not possible to rank the sealant materials according to the best clinical effectiveness in permanent molars of children and adolescents.
- In daily dental practice, child-related and tooth-related factors should be carefully weighed against the expected material-specific effects on caries prevention and the retention of different sealants. While optimum maintenance of dry working conditions favour the use of resin-based sealants, GI-based sealants may be preferred if proper isolation is questionable.
- However, future rigorously planned and well-implemented RCTs are needed to formulate reliable conclusions on the clinical effectiveness of sealants in primary teeth and permanent teeth of children and adolescents.

### Supplementary Information

Below is the link to the electronic supplementary material.

Supplementary file1 (DOCX 29 KB)

Supplementary file2 (DOCX 19 KB)

Supplementary file3 (DOCX 41 KB)

## Data Availability

The data of this umbrella review will be shared on considerable request by the authors.
