# Supplementary material for: Clinical effectiveness of pit and fissure sealants in primary and permanent teeth of children and adolescents: an umbrella review
Source: Eur Arch Paediatr Dent. 2024 Mar 15;25(3):289–315. doi: 10.1007/s40368-024-00876-9 (PMC11233332; doi:10.1007/s40368-024-00876-9)

PubMed Search 18.01.2023

Search number Query

Filters

Results

2,783

1

2

((pit OR ﬁssure) AND (sealant*)) AND (cari* OR cavit* OR decay*)

((pit[Title/Abstract] OR ﬁssure[Title/Abstract]) AND (sealant*[Title/Abstract])) AND (cari*[Title/Abstract] OR

cavit*[Title/Abstract] OR decay*[Title/Abstract])

1,023

3

4

5

6

7

((pit[Title/Abstract] OR ﬁssure[Title/Abstract]) AND (sealant*[Title/Abstract])) AND (cari*[Title/Abstract] OR

cavit*[Title/Abstract] OR decay*[Title/Abstract])

Systematic Review

50

49

(((pit[Title/Abstract] OR ﬁssure[Title/Abstract]) AND (sealant*[Title/Abstract])) AND (cari*[Title/Abstract] OR

cavit*[Title/Abstract] OR decay*[Title/Abstract]) AND (systematicreview[Filter])) AND (review[Title/Abstract])

(seal*[Title/Abstract]) AND (tooth[Title/Abstract] OR teeth[Title/Abstract] OR molar*[Title/Abstract] OR

premolar*[Title/Abstract])

7,550

1,231

45

((seal*[Title/Abstract]) AND (tooth[Title/Abstract] OR teeth[Title/Abstract] OR molar*[Title/Abstract] OR

premolar*[Title/Abstract])) AND (pit[Title/Abstract] OR ﬁssure[Title/Abstract])

((seal*[Title/Abstract]) AND (tooth[Title/Abstract] OR teeth[Title/Abstract] OR molar*[Title/Abstract] OR

premolar*[Title/Abstract])) AND (pit[Title/Abstract] OR ﬁssure[Title/Abstract])

Systematic Review

Systematic Review

8

9

(dental sealants[MeSH Terms]) AND (caries, dental[MeSH Terms])

(dental sealants[MeSH Terms]) AND (caries, dental[MeSH Terms])

1,943

71

1

0 ((dental sealants[MeSH Terms]) AND (caries, dental[MeSH Terms]) AND (systematicreview[Filter])) AND

69

(review[Title/Abstract])

1


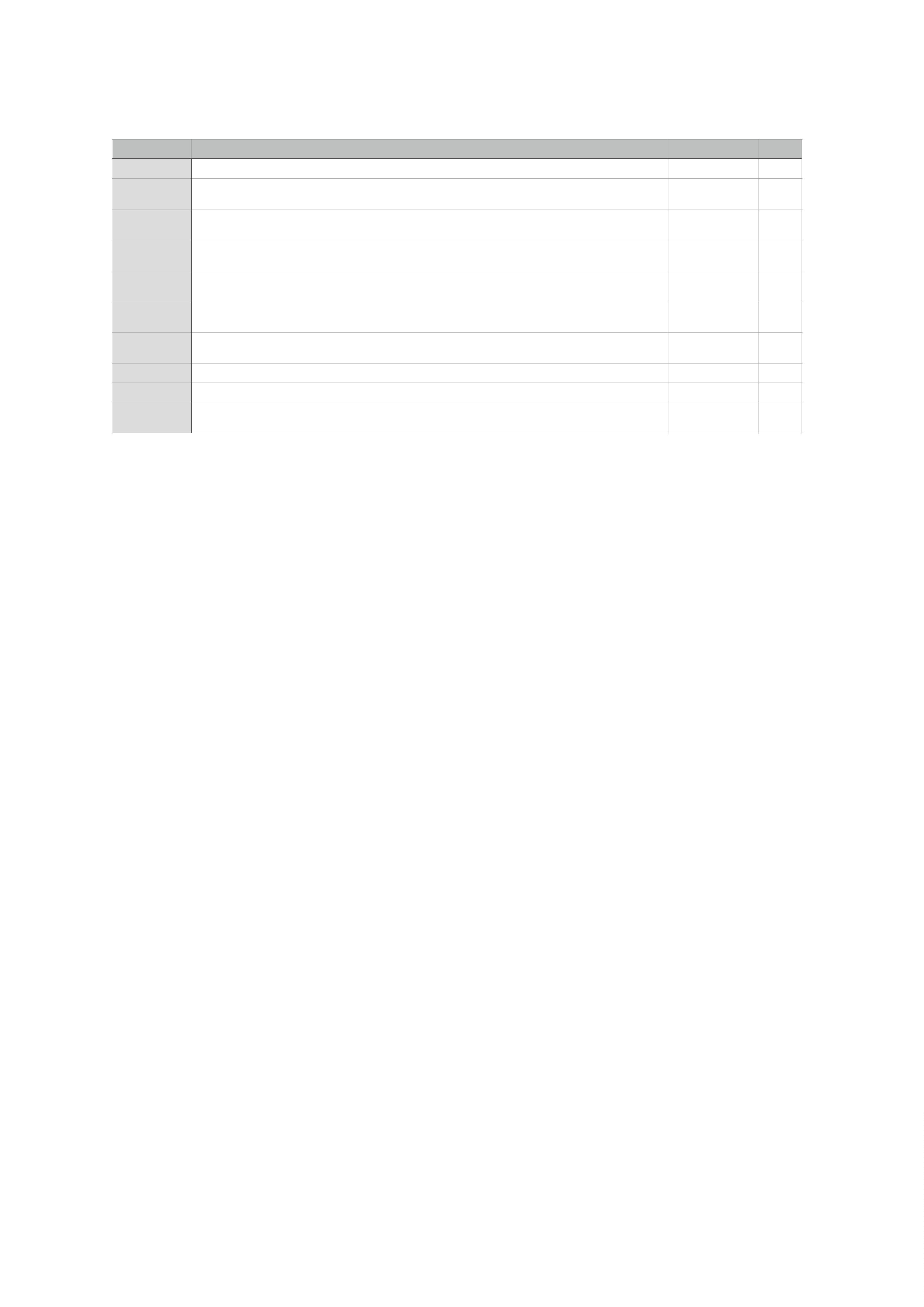

Supplement: Supplementary file 1 — Supplementary file1 (DOCX 29 KB) [file 40368_2024_876_MOESM1_ESM.docx]
