# Supplementary material for: Clinical effectiveness of pit and fissure sealants in primary and permanent teeth of children and adolescents: an umbrella review
Source: Eur Arch Paediatr Dent. 2024 Mar 15;25(3):289–315. doi: 10.1007/s40368-024-00876-9 (PMC11233332; doi:10.1007/s40368-024-00876-9)
Supplement: Supplementary file 2 — Supplementary file2 (DOCX 19 KB) [file 40368_2024_876_MOESM2_ESM.docx]

**Appendix 2**

**Exclusion of full texts from literature search**

| **Reasons for exclusion*** | **Authors and Year** | **Number of papers** |
| --- | --- | --- |
| **Does not meet inclusion criteria** *(e.g., data not split by follow-up, caries lesion extension > ICDAS-II 3, wrong age group, primary studies with follow-up < 12 mos included)* | Alharty et al. 2022; Alirezaei et al. 2018; Alsabek et al. 2021; Bader et al. 2001; Bagheri et al. 2022; Bagherian et al. 2016; Bagherian et al. 2018; Beiruti et al. 2006; Botton et al. 2016; Cabalen et al. 2022; de Amorim et al. 2018; Duangthip et al. 2015; Jafarzadeh et al. 2022; Kashbour et al. 2020; Kühnisch et al. 2020; Lam et al. 2020; Mickenautsch et al. 2011; Mickenautsch et al. 2013a; Mickenautsch et al. 2013b; Muller-Bolla et al. 2006; Pagano et al. 2020; Papageorgiou et al. 2017; Ramesh et al. 2020; Schwendicke et al. 2015; Taneja et al. 2020; Tedesco et al. 2022; Urquhart et al. 2019; Yengopal et al. 2010; Zhang et al. 2019 | **29** |
| **Wrong primary outcome**  *(e.g., does not evaluate sealant retention, caries incidence / progression, results only available for treatment combinations)* | Akinlotan et al. 2018; Griffin et al. 2017; Källestal et al. 2003; Kloukos et al. 2013; Leo et al. 2016; Marino et al. 2013 | **6** |
| **Former version of an updated systematic review** | Ahovuo-Saloranta et al. 2004; Ahovuo-Saloranta et al. 2008; Ahovuo-Saloranta et al. 2013; Ahovuo-Saloranta et al. 2016; Hiiri et al. 2006; Hiiri et al. 2010; Yengopal et al. 2009 | **7** |
| **Wrong type of study or publication**  *(e.g., practice guideline, report)* | AAPD Guideline 2018; Azarpazhooh & Main 2008a; Gugnani et al. 2018; Slayton et al. 2018 | **4** |
| Total: | | **46** |

* for studies with > 1 reason, only 1 reason will be given

**Exclusion of full texts from hand search**

| **Reasons for exclusion*** | **Authors and Year** | **Number of papers** |
| --- | --- | --- |
| **Not within the scope of this umbrella review** | Oong et al. 2008; Yengopal & Mickenautsch 2011; Yip & Smales 2002 | **3** |
| **Does not meet inclusion criteria** *(e.g., data not split by follow-up, caries lesion extension > ICDAS-II 3, wrong age group)* | Griffin et al. 2008 | **1** |
| **Wrong primary outcome**  *(e.g., does not evaluate sealant retention, caries incidence / progression, results only available for treatment combinations)* | Azarpazhooh & Main 2008b | **1** |
| **Missing information about quality assessment** | Condo et al. 2013 | **1** |
| **Former version of an updated systematic review** | Ahovuo-Saloranta et al. 1999 | **1** |
| **Wrong type of study or publication**  *(e.g., clinical trial, practice guideline, report)* | Baroudi & Rodrigues 2015; Beauchamp et al. 2008; Bojanini et al. 1976; Cvikl et al. 2018; Deery 1999; Donly & Garcia Godoy 2002; Feigal 2002; Kitchens 2005; Lewis & Morgan 1994; McLean & Wilson 1974; Morphis et al. 2000; Naaman et al. 2017; Neusser et al. 2014; Ripa 1993; Rock & Anderson 1982; Rock et al. 1978; Salem et al. 2014; Simonsen 1996; Simonsen 2002; Simonsen & Neal 2011; Swift 1988; Vrbic et al. 1983; Weintraub 1989; Weintraub 2001; Wright et al. 2016a | **25** |
| **Duplicate** | Bagherian et. al 2016; Wright et al. 2016b (either J Am Dent Assoc or Pediatr Dent 2016) | **2** |
| **Outdated materials included** | Llorda et al. 1993 | **1** |
| Total: | | **35** |

* for studies with > 1 reason, only 1 reason will be given
