# Supplementary material for: Clinical effectiveness of pit and fissure sealants in primary and permanent teeth of children and adolescents: an umbrella review
Source: Eur Arch Paediatr Dent. 2024 Mar 15;25(3):289–315. doi: 10.1007/s40368-024-00876-9 (PMC11233332; doi:10.1007/s40368-024-00876-9)
Supplement: Supplementary file 3 — Supplementary file3 (DOCX 41 KB) [file 40368_2024_876_MOESM3_ESM.docx]

**Appendix 3:** Citation matrix for overlap calculation of primary studies in umbrella review according to Pieper et al. (2014).

| **Overlap in umbrella review** | | **Included systematic reviews (c=7)** | | | | | | |  |
| --- | --- | --- | --- | --- | --- | --- | --- | --- | --- |
| **First author and year** | | **Ahovuo-Saloranta 2017** | **Li 2020** | **Mejare 2003** | **Mickenautsch  2016** | **Ramamurthy  2022** | **Rashed  2022** | **Wright  2016** |  |
|  |  |  |  |  |  |  |  |  |  |
| **Included primary studies (r=70)** | **Amin 2008** | X |  |  |  |  |  | X |  |
|  | **Antonson 2012** | X |  |  |  |  |  | X |  |
|  | **Arrow 1995** | X |  |  |  |  |  | X |  |
|  | **Baca 2007** |  |  |  |  | X |  |  |  |
|  | **Barja-Fidalgo 2009** | X |  |  | X |  |  | X |  |
|  | **Baseggio 2010** | X |  |  |  |  |  | X |  |
|  | **Bojanini 1976** | X |  |  |  |  |  | X |  |
|  | **Beiruti 2006** |  |  |  | X |  |  |  |  |
|  | **Bravo 1996** |  | X |  |  |  | X | X |  |
|  | **Bravo 1997a** |  | X |  |  |  | X |  |  |
|  | **Bravo 1997b** |  |  | X |  |  |  |  |  |
|  | **Bravo 2005** | X |  |  |  |  |  |  |  |
|  | **Brooks 1979** | X |  |  |  |  |  |  |  |
|  | **Chabadel 2021** |  |  |  |  | X |  |  |  |
|  | **Chadwick 2005** |  |  |  |  | X |  |  |  |
|  | **Charbeneau 1979** | X |  | X |  |  |  |  |  |
|  | **Chen 2012 (Zhang 2014)** | X |  |  | X |  |  | X |  |
|  | **Chen 2013** | X |  |  |  |  |  | X |  |
|  | **Chestnutt 2017** |  | X |  |  |  |  |  |  |
|  | **Corona 2005** |  |  |  |  | X |  |  |  |
|  | **De Luca-Fraga 2001** | X |  |  |  |  |  |  |  |
|  | **Dhar 2012** | X |  |  |  |  |  | X |  |
|  | **Erdogan 1987** | X |  |  |  |  |  | X |  |
|  | **Forss 1998** | X |  |  |  |  |  |  |  |
|  | **Ganesh 2006** | X |  |  |  | X |  | X |  |
|  | **Going 1977** |  |  | X |  |  |  |  |  |
|  | **Guler 2013** | X |  |  |  |  |  | X |  |
|  | **Güngör 2004** |  |  |  |  |  |  | X |  |
|  | **Haznedaroğlu 2016** |  |  |  |  |  |  | X |  |
|  | **Higson 1976** |  |  | X |  |  |  |  |  |
|  | **Hilgert 2015** |  |  |  | X |  |  |  |  |
|  | **Horowitz 1977** |  |  | X |  |  |  |  |  |
|  | **Hotuman 1998** |  |  |  |  | X |  |  |  |
|  | **Houpt 1983** |  |  |  |  |  |  | X |  |
|  | **Hunter 1988** | X |  |  |  |  |  |  |  |
|  | **Ji 2007** |  | X |  |  |  |  |  |  |
|  | **Joshi 2019** |  |  |  |  | X |  |  |  |
|  | **Karlzen-Reuterving 1995** | X |  |  |  |  |  |  |  |
|  | **Kervanto-Seppälä 2008** | X |  |  |  |  |  |  |  |
|  | **Leake 1976** |  |  | X |  |  |  |  |  |
|  | **Liu 2012** | X | X |  |  |  | X | X |  |
|  | **Liu 2014a** | X |  |  | X |  |  |  |  |
|  | **Liu 2014b** | X |  |  |  |  |  |  |  |
|  | **Mertz-Fairhurst 1984** |  |  |  |  |  |  | X |  |
|  | **Mills 1993** | X |  |  |  |  |  |  |  |
|  | **Muller-Bolla 2013** | X |  |  |  |  |  |  |  |
|  | **Oba 2009** |  |  |  | X |  |  |  |  |
|  | **Pardi 2005** | X |  |  |  |  |  | X |  |
|  | **Pereira 2003** |  |  | X |  |  |  | X |  |
|  | **Poulsen 1979** |  |  | X |  |  |  |  |  |
|  | **Poulsen 2001** | X |  |  |  |  |  |  |  |
|  | **Raadal 1984** |  | X | X |  |  |  |  |  |
|  | **Raadal 1996** | X |  |  |  |  |  |  |  |
|  | **Ren 2011** |  |  |  |  | X |  |  |  |
|  | **Reisbick 1982** | X |  |  |  |  |  |  |  |
|  | **Richardson 1978** | X |  |  |  |  |  |  |  |
|  | **Richardson 1980** |  |  | X |  |  |  | X |  |
|  | **Rock 1978** | X |  |  |  |  |  |  |  |
|  | **Rock 1996** | X |  |  |  |  |  |  |  |
|  | **Salem 2014** |  | X |  |  |  | X |  |  |
|  | **Sheykholeslam 1978** | X |  |  |  |  |  |  |  |
|  | **Sipahier 1995** | X |  |  |  |  |  |  |  |
|  | **Songpaisan 1995** | X |  | X |  |  |  |  |  |
|  | **Splieth 2001** |  |  |  |  |  |  | X |  |
|  | **Stephen 1978** |  |  | X |  |  |  |  |  |
|  | **Tagliaferro 2011** | X | X |  |  |  |  | X |  |
|  | **Tang 2014** | X |  |  |  |  |  |  |  |
|  | **Thylstrup 1978** |  |  | X |  |  |  |  |  |
|  | **Unal 2015** |  |  |  |  | X |  |  |  |
|  | **Williams 1996** | X |  |  |  |  |  |  |  |
|  | **Total number of primary studies** | 38 | 8 | 13 | 6 | 9 | 4 | 23 |  |
| **Number of included primary studies (double counting) N=101** | | | | | | | | |  |

| *N = 101* | $CCA=(N-r)/(r*c-r)$   \|  \| \| --- \| |  |  |  |  |
| --- | --- | --- | --- | --- | --- | --- |
| *c = 7* |  |  |  |  |  |
| *r = 70* | ***CCA = 0.074* → *7.4 %* → *moderate overlap according to Pieper et al. (2014)*** | | | |  |
|  |  |  |  |  |  |

**References of the included primary studies**

1. Amin HE. Clinical and antibacterial effectiveness of three different sealant materials. J Dent Hyg. 2008; 82(5): 45.
2. Antonson SA, Antonson DE, Brener S, Crutchfield J, Larumbe J, Michaud C, et al. Twenty-four month clinical evaluation of fissure sealants on partially erupted permanent first molars: glass ionomer versus resin-based sealant. J Am Dent Assoc. 2012; 143(2): 115-122. doi:10.14219/jada.archive.2012.0121
3. Arrow P, Riordan PJ. Retention and caries preventive effects of a GIC and a resin-based fissure sealant. Community Dent Oral Epidemiol. 1995; 23(5): 282-285. doi:10.1111/j.1600-0528.1995.tb00249.x
4. Baca P, Bravo M, Baca AP, Jimenez A, Gonzalez-Rodriguez MP. Retention of three fissure sealants and a dentin bonding system used as fissure sealant in caries prevention: 12-month follow-up results. Med Oral Patol Oral Cir Bucal. 2007; 12(6): E459-463.
5. Barja-Fidalgo F, Maroun S, de Oliveira BH. Effectiveness of a glass ionomer cement used as a pit and fissure sealant in recently erupted permanent first molars. J Dent Child (Chic). 2009; 76(1): 34-40.
6. Baseggio W, Naufel FS, Davidoff DC, Nahsan FP, Flury S, Rodrigues JA. Caries-preventive efficacy and retention of a resin-modified glass ionomer cement and a resin-based fissure sealant: a 3-year split-mouth randomised clinical trial. Oral Health Prev Dent. 2010; 8(3): 261-268.
7. Beiruti N, Frencken JE, van't Hof MA, Taifour D, van Palenstein Helderman WH. Caries-preventive effect of a one-time application of composite resin and glass ionomer sealants after 5 years. Caries Res. 2006; 40(1): 52-59. doi:10.1159/000088907
8. Bojanini J, Garces H, McCune RJ, Pineda A. Effectiveness of pit and fissure sealants in the prevention of caries. J Prev Dent. 1976; 3(6): 31-34.
9. Bravo M, Baca P, Llodra JC, Osorio E. A 24-month study comparing sealant and fluoride varnish in caries reduction on different permanent first molar surfaces. J Public Health Dent. 1997; 57(3): 184-186. doi:10.1111/j.1752-7325.1997.tb02972.x
10. Bravo M, García‐Anllo I, Baca P, Llodra JC. A 48‐month survival analysis comparing sealant (Delton) with fluoride varnish (Duraphat) in 6‐to 8‐year‐old children. Community Dent Oral Epidemiol. 1997; 25(3): 247-250.
11. Bravo M, Llodra JC, Baca P, Osorio E. Effectiveness of visible light fissure sealant (Delton) versus fluoride varnish (Duraphat): 24-month clinical trial. Community Dent Oral Epidemiol. 1996; 24(1): 42-46. doi:10.1111/j.1600-0528.1996.tb00811.x
12. Bravo M, Montero J, Bravo JJ, Baca P, Llodra JC. Sealant and fluoride varnish in caries: a randomized trial. J Dent Res. 2005; 84(12): 1138-1143. doi:10.1177/154405910508401209
13. Brooks JD, Mertz-Fairhurst EJ, Della-Giustina VE, Williams JE, Fairhurst CW. A comparative study of two pit and fissure sealants: two-year results in Augusta, Ga. J Am Dent Assoc. 1979; 98(5): 722-725. doi:10.14219/jada.archive.1979.0149
14. Chabadel O, Veronneau J, Montal S, Tramini P, Moulis E. Effectiveness of pit and fissure sealants on primary molars: A 2-yr split-mouth randomized clinical trial. Eur J Oral Sci. 2021; 129(1): e12758. doi:10.1111/eos.12758
15. Chadwick BL, Treasure ET, Playle RA. A randomised controlled trial to determine the effectiveness of glass ionomer sealants in pre-school children. Caries Res. 2005; 39(1): 34-40. doi:10.1159/000081654
16. Charbeneau GT, Dennison JB. Clinical success and potential failure after single application of a pit and fissure sealant: a four-year report. J Am Dent Assoc. 1979; 98(4): 559-564. doi:10.14219/jada.archive.1979.0112
17. Chen X, Du MQ, Fan MW, Mulder J, Huysmans MC, Frencken JE. Caries-preventive effect of sealants produced with altered glass-ionomer materials, after 2 years. Dent Mater. 2012; 28(5): 554-560. doi:10.1016/j.dental.2012.01.001
18. Chen X, Liu X. Clinical comparison of Fuji VII and a resin sealant in children at high and low risk of caries. Dent Mater J. 2013; 32(3): 512-518. doi:10.4012/dmj.2012-300
19. Chestnutt IG, Hutchings S, Playle R, Morgan-Trimmer S, Fitzsimmons D, Aawar N, et al. Seal or Varnish? A randomised controlled trial to determine the relative cost and effectiveness of pit and fissure sealant and fluoride varnish in preventing dental decay. Health Technol Assess. 2017; 21(21): 1-256. doi:10.3310/hta21210
20. Corona SA, Borsatto MC, Garcia L, Ramos RP, Palma-Dibb RG. Randomized, controlled trial comparing the retention of a flowable restorative system with a conventional resin sealant: one-year follow up. Int J Paediatr Dent. 2005; 15(1): 44-50. doi:10.1111/j.1365-263X.2005.00605.x
21. de Luca-Fraga LR, Pimenta LA. Clinical evaluation of glass-ionomer/resin-based hybrid materials used as pit and fissure sealants. Quintessence Int. 2001; 32(6): 463-468.
22. Dhar V, Chen H. Evaluation of resin based and glass ionomer based sealants placed with or without tooth preparation-a two year clinical trial. Pediatr Dent. 2012; 34(1): 46-50.
23. Erdogan B, Alaçam T. Evaluation of a chemically polymerized pit and fissure sealant: results after 4.5 years. J Paediatr Dent. 1987; 3: 11-13.
24. Forss H, Halme E. Retention of a glass ionomer cement and a resin-based fissure sealant and effect on carious outcome after 7 years. Community Dent Oral Epidemiol. 1998; 26(1): 21-25.
25. Ganesh M, Tandon S. Clinical evaluation of FUJI VII sealant material. J Clin Pediatr Dent. 2006; 31(1): 52-57. doi:10.17796/jcpd.31.1.w8532743883hw64n
26. Going RE, Haugh LD, Grainger DA, Conti AJ. Four-year clinical evaluation of a pit and fissure sealant. J Am Dent Assoc. 1977; 95(5): 972-981. doi:10.14219/jada.archive.1977.0163
27. Guler C, Yilmaz Y. A two-year clinical evaluation of glass ionomer and ormocer based fissure sealants. J Clin Pediatr Dent. 2013; 37(3): 263-267. doi:10.17796/jcpd.37.3.38761uwwm7kpj616
28. Güngör HC, Altay N, Alpar R. Clinical evaluation of a polyacid-modified resin composite-based fissure sealant: two-year results. Oper Dent. 2004; 29(3): 254-260.
29. Haznedaroglu E, Guner S, Duman C, Mentes A. A 48-month randomized controlled trial of caries prevention effect of a one-time application of glass ionomer sealant versus resin sealant. Dent Mater J. 2016; 35(3): 532-538. doi:10.4012/dmj.2016-084
30. Higson JF. Caries prevention in first permanent molars by fissure sealing. A 2-year study in 6--8-year-old children. J Dent. 1976; 4(5): 218-222. doi:10.1016/0300-5712(76)90051-8
31. Hilgert LA, Leal SC, Mulder J, Creugers NH, Frencken JE. Caries-preventive Effect of Supervised Toothbrushing and Sealants. J Dent Res. 2015; 94(9): 1218-1224. doi:10.1177/0022034515592857
32. Horowitz HS, Heifetz SB, Poulsen S. Retention and effectiveness of a single application of an adhesive sealant in preventing occlusal caries: final report after five years of a study in Kalispell, Montana. J Am Dent Assoc. 1977; 95(6): 1133-1139. doi:10.14219/jada.archive.1977.0201
33. Hotuman E, Rolling I, Poulsen S. Fissure sealants in a group of 3-4-year-old children. Int J Paediatr Dent. 1998; 8(2): 159-160. doi:10.1046/j.1365-263x.1998.00076.x
34. Houpt M, Shey Z. The effectiveness of a fissure sealant after six years. Pediatr Dent. 1983; 5(2): 104-106.
35. Hunter PB. A study of pit and fissure sealing in the School Dental Service. N Z Dent J. 1988; 84(375): 10-12.
36. Ji PH, Xu QL, Ba Y. [Clinical evaluation of fluor protector and glass-ionomer cement used as pit and fissure sealant for preventing pit and fissure caries in children]. Shanghai Kou Qiang Yi Xue. 2007; 16(4): 374-376.
37. Joshi S, Sandhu M, Sogi HPS, Garg S, Dhindsa A. Split-mouth Randomised Clinical Trial on the Efficacy of GIC Sealant on Occlusal Surfaces of Primary Second Molar. Oral Health Prev Dent. 2019; 17(1): 17-24. doi:10.3290/j.ohpd.a41979
38. Karlzén-Reuterving G, van Dijken JW. A three-year follow-up of glass ionomer cement and resin fissure sealants. ASDC J Dent Child. 1995; 62(2): 108-110.
39. Kervanto‐Seppälä S, Lavonius E, Pietilä I, Pitkäniemi J, Meurman JH, Kerosuo E. Comparing the caries‐preventive effect of two fissure sealing modalities in public health care: a single application of glass ionomer and a routine resin‐based sealant programme. A randomized split‐mouth clinical trial. International Journal of Paediatr Dent. 2008; 18(1): 56-61.
40. Leake JL, Martinello BP. A four year evaluation of a a fissure sealant in a public health setting. Dent J. 1976; 42(8): 409-415.
41. Liu BY, Lo EC, Chu CH, Lin HC. Randomized trial on fluorides and sealants for fissure caries prevention. J Dent Res. 2012; 91(8): 753-758. doi:10.1177/0022034512452278
42. Liu BY, Xiao Y, Chu CH, Lo EC. Glass ionomer ART sealant and fluoride-releasing resin sealant in fissure caries prevention--results from a randomized clinical trial. BMC Oral Health. 2014; 14: 54. doi:10.1186/1472-6831-14-54
43. Liu Y, Rong W, Zhao X, Wang M, Jiang Q, Wang W. [Caries prevention effect of resin based sealants and glass ionomor sealants]. Zhonghua Kou Qiang Yi Xue Za Zhi. 2014; 49(4): 199-203.
44. Mertz-Fairhurst EJ, Fairhurst CW, Williams JE, Della-Giustina VE, Brooks JD. A comparative clinical study of two pit and fissure sealants: 7-year results in Augusta, GA. J Am Dent Assoc. 1984; 109(2): 252-255. doi:10.14219/jada.archive.1984.0347
45. Mills RW, Ball IA. A clinical trial to evaluate the retention of a silver cermet-ionomer cement used as a fissure sealant. Oper Dent. 1993; 18(4): 148-154.
46. Muller-Bolla M, Lupi-Pegurier L, Bardakjian H, Velly AM. Effectiveness of school-based dental sealant programs among children from low-income backgrounds in France: a pragmatic randomized clinical trial. Community Dent Oral Epidemiol. 2013; 41(3): 232-241. doi:10.1111/cdoe.12011
47. Oba AA, Dulgergil T, Sonmez IS, Dogan S. Comparison of caries prevention with glass ionomer and composite resin fissure sealants. J Formos Med Assoc. 2009; 108(11): 844-848. doi:10.1016/S0929-6646(09)60415-0
48. Pardi V, Pereira AC, Ambrosano GM, Meneghim Mde C. Clinical evaluation of three different materials used as pit and fissure sealant: 24-months results. J Clin Pediatr Dent. 2005; 29(2): 133-137. doi:10.17796/jcpd.29.2.e44h17387x324345
49. Pereira AC, Pardi V, Mialhe FL, Meneghim Mde C, Ambrosano GM. A 3-year clinical evaluation of glass-ionomer cements used as fissure sealants. Am J Dent. 2003; 16(1): 23-27.
50. Poulsen S, Beiruti N, Sadat N. A comparison of retention and the effect on caries of fissure sealing with a glass-ionomer and a resin-based sealant. Community Dent Oral Epidemiol. 2001; 29(4): 298-301. doi:10.1034/j.1600-0528.2001.290409.x
51. Poulsen S, Thylstrup A, Christensen PF, Ishoy. Evaluation of a pit- and fissure-sealing program in a public dental health service after 2 years. Community Dent Oral Epidemiol. 1979; 7(3): 154-157. doi:10.1111/j.1600-0528.1979.tb01205.x
52. Raadal M, Laegreid O, Laegreid KV, Hveem H, Korsgaard EK, Wangen K. Fissure sealing of permanent first molars in children receiving a high standard of prophylactic care. Community Dent Oral Epidemiol. 1984; 12(2): 65-68. doi:10.1111/j.1600-0528.1984.tb01414.x
53. Raadal M, Utkilen AB, Nilsen OL. Fissure sealing with a light-cured resin-reinforced glass-ionomer cement (Vitrebond) compared with a resin sealant. Int J Paediatr Dent. 1996; 6(4): 235-239. doi:10.1111/j.1365-263x.1996.tb00251.x
54. Reisbick MH, Thanos CE, Berson RB, Goldstein CM. Benefit from sealants in a moderately fluoridated community. CDA J. 1982; 10(1): 53-56.
55. Ren F, Liu J-p, Huang S-h, Li Y-r, Fan W-h, Chen X-c, et al. Application of glass ionomer and light-cured resin sealant to the pit and fissure of deciduous teeth. Chinese J Tissue Eng Res. 2011; 15(38): 7165.
56. Richardson AS, Gibson GB, Waldman R. The effectiveness of a chemically polymerized sealant: four-year results. Pediatr Dent. 1980; 2(1): 24-26.
57. Richardson AS, Waldman R, Gibson GB, Vancouver BC. The effectiveness of a chemically polymerized sealant in preventing occlusal caries: two year results. Dent J. 1978; 44(6): 269-272.
58. Rock WP, Foulkes EE, Perry H, Smith AJ. A comparative study of fluoride-releasing composite resin and glass ionomer materials used as fissure sealants. J Dent. 1996; 24(4): 275-280. doi:10.1016/0300-5712(95)00061-5
59. Rock WP, Gordon PH, Bradnock G. The effect of operator variability and patient age on the retention of fissure sealant resin. Br Dent J. 1978; 145(3): 72-75. doi:10.1038/sj.bdj.4804121
60. Salem K, Shahsavari F, Kazemnejad E, Poorhabibi Z. Pit and fissure sealant versus fluoride varnish in prevention of occlusal caries. J Dentomaxillofacial. 2014; 3(1): 46-47.
61. Sheykholeslam Z, Houpt M. Clinical effectiveness of an autopolymerized fissure sealant after 2 years. Community Dent Oral Epidemiol. 1978; 6(4): 181-184. doi:10.1111/j.1600-0528.1978.tb01146.x
62. Sipahier M, Ulusu T. Glass-ionomer--silver-cermet cements applied as fissure sealants II. Clinical evaluation. Quintessence Int. 1995; 26(1).
63. Songpaisan Y, Bratthall D, Phantumvanit P, Somridhivej Y. Effects of glass ionomer cement, resin-based pit and fissure sealant and HF applications on occlusal caries in a developing country field trial. Community Dent Oral Epidemiol. 1995; 23(1): 25-29. doi:10.1111/j.1600-0528.1995.tb00193.x
64. Splieth C, Förster M, Meyer G. Additional caries protection by sealing permanent first molars compared to fluoride varnish applications in children with low caries prevalence: 2-year results. Eur J Paediatr Dent. 2001; 2(3): 133-137.
65. Stephen KW. A four-year fissure sealing study in fluoridated and non-fluoridated Galloway. Health Bull (Edinb). 1978; 36(3): 138-145.
66. Tagliaferro EP, Pardi V, Ambrosano GM, Meneghim Mde C, da Silva SR, Pereira AC. Occlusal caries prevention in high and low risk schoolchildren. A clinical trial. Am J Dent. 2011; 24(2): 109-114.
67. Tang LH, Shi L, Yuan S, Lv J, Lu HX. [Effectiveness of 3 different methods in prevention of dental caries in permanent teeth among children]. Shanghai Kou Qiang Yi Xue. 2014; 23(6): 736-739.
68. Thylstrup A, Poulsen S. Retention and effectiveness of a chemically polymerized pit and fissure sealant after 2 years. Scand J Dent Res. 1978; 86(1): 21-24. doi:10.1111/j.1600-0722.1978.tb00603.x
69. Unal M, Oznurhan F, Kapdan A, Durer S. A comparative clinical study of three fissure sealants on primary teeth: 24-month results. J Clin Pediatr Dent. 2015; 39(2): 113-119. doi:10.17796/jcpd.39.2.h247471176596757
70. Williams B, Laxton L, Holt RD, Winter GB. Fissure sealants: a 4-year clinical trial comparing an experimental glass polyalkenoate cement with a bis glycidyl methacrylate resin used as fissure sealants. Br Dent J. 1996; 180(3): 104-108. doi:10.1038/sj.bdj.4808989
